# Comparative Genomic Analysis Reveals a Diverse Repertoire of Genes Involved in Prokaryote-Eukaryote Interactions within the *Pseudovibrio* Genus

**DOI:** 10.3389/fmicb.2016.00387

**Published:** 2016-03-30

**Authors:** Stefano Romano, Antonio Fernàndez-Guerra, F. Jerry Reen, Frank O. Glöckner, Susan P. Crowley, Orla O'Sullivan, Paul D. Cotter, Claire Adams, Alan D. W. Dobson, Fergal O'Gara

**Affiliations:** ^1^BIOMERIT Research Centre, University College CorkCork, Ireland; ^2^Oxford e-Research Centre, University of OxfordOxford, UK; ^3^Microbial Genomics and Bioinformatics Research Group, Max Planck Institute for Marine MicrobiologyBremen, Germany; ^4^Jacobs University Bremen gGmbHBremen, Germany; ^5^Teagasc Food Research CentreFermoy, Ireland; ^6^APC Microbiome InstituteCork, Ireland; ^7^School of Microbiology, University College CorkCork, Ireland; ^8^Environmental Research Institute, University College CorkCork, Ireland; ^9^School of Biomedical Sciences, Curtin Health Innovation Research Institute, Curtin UniversityPerth, WA, Australia

**Keywords:** *Pseudovibrio*, comparative genomics, symbiosis, secretion systems, toxins, core-genome, phylogenesis

## Abstract

Strains of the *Pseudovibrio* genus have been detected worldwide, mainly as part of bacterial communities associated with marine invertebrates, particularly sponges. This recurrent association has been considered as an indication of a symbiotic relationship between these microbes and their host. Until recently, the availability of only two genomes, belonging to closely related strains, has limited the knowledge on the genomic and physiological features of the genus to a single phylogenetic lineage. Here we present 10 newly sequenced genomes of *Pseudovibrio* strains isolated from marine sponges from the west coast of Ireland, and including the other two publicly available genomes we performed an extensive comparative genomic analysis. Homogeneity was apparent in terms of both the orthologous genes and the metabolic features shared amongst the 12 strains. At the genomic level, a key physiological difference observed amongst the isolates was the presence only in strain *P. axinellae* AD2 of genes encoding proteins involved in assimilatory nitrate reduction, which was then proved experimentally. We then focused on studying those systems known to be involved in the interactions with eukaryotic and prokaryotic cells. This analysis revealed that the genus harbors a large diversity of toxin-like proteins, secretion systems and their potential effectors. Their distribution in the genus was not always consistent with the phylogenetic relationship of the strains. Finally, our analyses identified new genomic islands encoding potential toxin-immunity systems, previously unknown in the genus. Our analyses shed new light on the *Pseudovibrio* genus, indicating a large diversity of both metabolic features and systems for interacting with the host. The diversity in both distribution and abundance of these systems amongst the strains underlines how metabolically and phylogenetically similar bacteria may use different strategies to interact with the host and find a niche within its microbiota. Our data suggest the presence of a sponge-specific lineage of *Pseudovibrio*. The reduction in genome size and the loss of some systems potentially used to successfully enter the host, leads to the hypothesis that *P. axinellae* strain AD2 may be a lineage that presents an ancient association with the host and that may be vertically transmitted to the progeny.

## Introduction

The genus *Pseudovibrio* consists of five type strains: *P. denitrificans* and *P. japonicus*, isolated from coastal seawater, *P. ascidiaceicola* isolated from a marine tunicate, *P. axinellae* isolated from a marine sponge, and *P. hongkongensis* isolated from a marine flatworm (Bondarev et al., 2013; Xu et al., 2015). All type strains share similar physiological traits, being described as marine, heterotrophic, facultative anaerobic bacteria, capable of denitrifying and fermenting different substrates. Although just five type strains have been described, bacteria belonging to this genus have been detected from a wide variety of sources, as both free leaving and associated with marine invertebrates, especially corals, and sponges (Bondarev et al., 2013). In addition to being recurrently identified in the sponge microbiota, *Pseudovibrio* related strains have also been reported to be dominant in the culturable bacterial fraction of different marine sponges (Webster and Hill, 2001; Muscholl-Silberhorn et al., 2007; Bauvais et al., 2015) and, indeed, it has been suggested that a symbiotic relationship might exist between bacteria of the *Pseudovibrio* genus and these marine invertebrates (Taylor et al., 2007). In support of this hypothesis bacteria belonging to the *Pseudovibrio* genus have been found to be the most abundant prokaryotes associated with larvae of the sponge *Mycale laxissima*, indicating vertical transmission (i.e. direct transmission from the parental line to the progeny) of these microbes within their hosts (Enticknap et al., 2006).

The nature of the symbiosis, defined here as: “an association between dissimilar organisms” (de Bary, 1879), between *Pseudovibrio* and marine invertebrates is still unclear, due to the current absence of experimental evidence that could help unravel whether these bacteria are mutualists/commensalists or pathogens/parasites. There is only one report linking *Pseudovibrio*-related bacteria to bleached scleractinian corals (Moreira et al., 2013) and in general bacteria related to *Pseudovibrio* have only been isolated from healthy sponges and other metazoa. Moreover, recent evidence suggests that *Pseudovibrio*-related strains decrease drastically in abundance with the decline in sponge health, being completely absent from diseased sponges (Sweet et al., 2015). These findings appear to indicate that *Pseudovibrio* do not harm the host, and may even be required for its survival and its health (Webster and Hill, 2001).

Generally, obligate symbiotic bacteria (pathogens or mutualists) undergo a progressive reduction in their genome size. This is particularly true of those that are vertically transmitted, and which have an ancient symbiotic relationship with their hosts. As part of this process virulence genes such as type III secretion systems (T3SS) and its effectors are lost (Dale and Moran, 2006; McCutcheon and Moran, 2012). The general idea is that the genetic characteristics of symbionts reflect their mechanism of transmission. Large genomes are generally an indication of ecological versatility, which is important for bacteria that have both free-living and host-associated lifestyles and that are environmentally (horizontally) acquired by their hosts (Konstantinidis and Tiedje, 2004; Goffredi et al., 2014). This mode of transmission is reflected in the presence of genes encoding systems for interacting with both the extracellular matrices and the cells of the host (Dale and Moran, 2006; Hentschel et al., 2012; Goffredi et al., 2014). In fact, the recent increase in bacterial genome data has revealed that secretion systems, such as the type III, IV, and VI, which have been historically associated with pathogenic strains, are in fact widespread in both symbiotic and free living bacteria (Dale and Moran, 2006; Persson et al., 2009). These secretion systems are structures commonly used by bacteria to directly deliver into target cells specific protein “effectors” that are able to generate specific alteration within the recipient organisms, allowing the delivering cells to outcompete other bacteria or to avoid digestion by the host, permitting successful colonization (Costa et al., 2015).

Recently, physiological and genomic analyses performed on the two strains related to *P. denitrificans* for which genome sequences are available, FO-BEG1 and JE062, showed that these bacteria are characterized by a large genome size (>5.7 Mbp), are well-adapted to thrive under different environmental conditions, and harbor some systems that could play a role in the interaction with their eukaryotic hosts (Bondarev et al., 2013; Romano et al., 2014, 2015; Schwedt et al., 2015). Altogether these data suggest that these bacteria are mainly facultative symbionts of marine invertebrates, and still maintain a great metabolic versatility that allows them to thrive under the periodic nutrient fluctuations observed in the open ocean.

Even though these studies provided an insight into the physiological and genomic features of this understudied genus, they were conducted on closely related strains affiliated to *Pseudovibrio denitrificans*. Therefore, there is still a lack of knowledge with respect to the genomic and physiological features of this genus, and the mechanisms potentially used by *Pseudovibrio* strains to thrive in association with their eukaryotic hosts are still poorly understood. Here we present 10 newly sequenced *Pseudovibrio* genomes, obtained from strains isolated from marine sponges collected from the west coast of Ireland (O'Halloran et al., 2011). We performed a comparative genomic analysis including also the two publicly available *Pseudovibrio* FO-BEG1 and JE062 genomes, and we describe the general physiological features of these bacteria, focusing on the traits that are likely to be fundamental for these strains to establish and maintain a successful relationship with the host and secure a place in its microbiota.

## Materials and methods

### Genome sequencing and assembly

The genomes of strain AD13, AD14, AD26, AD37, AD46, and AD5 were sequenced using a combination of Roche GS FLX 454 and Illumina MiSeq sequencer (Teagasc Sequencing Centre, Moorepark, Ireland). All reads were visually inspected using the FASTQC tool v.0.11.3 (Andrews, 2010). 454 sequences reads were initially *de-novo* assembled using Newbler v 2.0.01.14 with default parameters. Subsequently the obtained 454 contigs were combined and assembled with MiSeq reads using SPADES v3.1.1 (Bankevich et al., 2012), employing varying kmers of 41, 55, 59, 71, 99, 127, and MismatchCorrector for improved output (Li and Durbin, 2009). Resulting contigs were inspected for misassembles with AMOS v3.1.0 and HAWKEYE v2.0 (Schatz et al., 2013). The genomes of strain AD2, WM33 (formally AD30), W64 (formally AD8), and W74 (formally AD15) were sequenced using an Illumina HiSeq sequencer. Reads were dynamically trimmed and filtered by length using the lowest median value of each paired read by the SolexaQA software suite release 2.0 (Cox et al., 2010). After the quality assessment, the resulting reads were digitally normalized by the khmer software package (Brown et al., 2012). Two different passes were conducted with the *k*-mer size (*k*) = 20, and with two different coverage thresholds (*C*) = 20 and *C* = 5. To find the best parameters that maximizes the N50 and the length of the largest contig for Velvet assembler (Zerbino and Birney, 2008), we used VelvetOptimiser v2.2.5 (VelvetOptimiser^1^). Using the *de-novo* assembly returned by the VelvetOptimiser, the mean and standard deviation values were estimated for the insert length mapping the reads with Bowtie2 v2.0.6 (Langmead and Salzberg, 2012) against the assembly. The final assembly was performed using Velvet v1.2.10 in combination with all the parameters provided by VelvetOptimiser and with the estimated insert length measurements.

### Genome annotation, analysis of the core-genome, and phylogenetic analyses

Genomes were annotated using Prokka v1.10 (Seemann, 2014), and their completeness was estimated using CheckM v1.0.3, considering the marker genes uniquely present in the family *Rhodobacteraceae* (Parks et al., 2015). ANI and Tetra values were calculated using the JSpecies v1.2.1 program (Richter and Rosselló-Móra, 2009). KEGG modules were identified in each genome submitting the encoded proteins (proteomes) to BLASTKOALA v1.3 (Kanehisa et al., 2012). Proteins were then assigned to COG using the COGsoft v2.1, and the updated version of the COG database (Galperin et al., 2015). Orthologous gene families were calculated at the protein level using the three algorithms (BDBH; COG; OrthoMCL) implemented in GET_HOMOLOGUES v1.0, using a minimum coverage in the Blast pairwise alignments of 50%. As implemented in GET_HOMOLOGUES, consensus core-genome was obtained intersecting the prediction performed using all three algorithm.

Proteins belonging to the consensus core genomes were aligned using MUSCLE v3.8.31 (Edgar, 2004) and used as reference for the alignment of the respective genes. For a randomly selected subset of genes, substitution saturation was estimated using the APE package (Paradis et al., 2004) in the R program v3.2.0 (R Core Team, 2014), with and without considering the third nucleotide of each codon. Considering the low level of saturation the whole nucleotide sequences were used for phylogenetic inference. A maximum likelihood phylogenetic tree was constructed with RAxML v8.0.26 (Stamatakis, 2014), using the partition identified with PartitionFinder v1.1.1 (Lanfear et al., 2012), the position weights calculated using ZORRO (Wu et al., 2012), the GTRGAMMA option as model of nucleotide substitution, and allowing 1000 bootstrap repetitions. Phylogenetic analysis based on the 16S rRNA genes was performed adding the sequences of the strains used in this work and the sequence of the new type strain *P. hongkongensis* (Xu et al., 2015) into the ARB database used by Bondarev et al. (2013). Sequences were aligned using the SINA v1.3.0 (Silva Incremental Aligner; Pruesse et al., 2012) integrated into ARB v6.0.2 (Ludwig et al., 2004). Partial 16S and redundant sequences were manually removed. In order to account for differences in length amongst the sequences in the dataset 0, 30, and 50% position variability filters were created considering only nucleotide positions between 36 and 1464, according with *E. coli* numbering. Maximum parsimony and maximum likelihood (RAxML) trees were constructed using each of the above mentioned filters, and a consensus tree was built using the implemented feature in ARB.

For the subsequent analyses of the virulence related genes all 12 proteomes were scanned with the *PfamScan* script (Sanger, Pfamscan^2^) and *hmmscan* (HMMER3.0; Eddy, 2008) to identify Pfam-A (Pfam release 27.0; Finn et al., 2014) and TIGRFAM domains (TIGRFAM release 15.0; Haft et al., 2013), respectively. Finally, proteins of all genomes were hierarchically clustered using h-cd-hit (Huang et al., 2010) with threshold identities of 90, 80, and 70% for the three clustering steps, and allowing a maximum length difference amongst proteins of 75 amino acid. Hereafter, the obtained protein cluster will be referred to as cd-hit protein-clusters.

### Nitrate assimilation

*Pseudovibrio* isolates were incubated overnight at 23°C in marine broth. Overnight cultures were washed twice and resuspended in PBS. Washed cultures were inoculated at a starting optical density (OD_600*nm*_) of 0.01 into 20 ml of a modified CM medium (Bondarev et al., 2013) where NH_4_ was replaced with KNO_3_ as sole nitrogen source. CM KNO_3_ medium: 0.6% (w/v) Tris-HCl, 0.144% (w/v) MgCl_2_, 3% (w/v) NaCl, 0.2% (w/v) K_2_SO_4_, 0.1% (w/v) KNO_3_, pH adjusted to 7.5 with 1 M NaOH. 10 mM Glucose, 1 mM K_2_HPO_4_, 0.1% (v/v) mixed metals (Teknova 1000x).

### Annotation of the type III secretion system (T3SS)

Proteins belonging to COGs related to the T3SS were recovered and combined with proteins containing Pfam and TIGRFAM domains characteristic for the T3SS (Persson et al., 2009). In order to verify whether all structural components of the T3SS were identified, the obtained proteins were annotated using the KEGGMAPPER interface v2.5 (Kanehisa et al., 2012). The KEGG modules referring to the T3SS were then considered. For all the orthologous groups (KOs) that were not identified amongst the *Pseudovibrio* query sequences, subsets of proteins belonging to *Escherichia* sp., *Yersinia* sp., and *Salmonella* sp. were recovered from the KEGG database and used to build HMM models. These models were then used to scan (*hmmscan*) the 12 *Pseudovibrio* proteomes with a domain *E*-value ≤ 10^−6^. A representative genomic region containing adjacent genes encoding for the identified T3SS structural component (FO-BEG1_3657-03696) was then used as query to look for homologous regions in the 12 *Pseudovibrio* genomes with MultiGeneBlast v1.1.0 (Medema et al., 2013). Proteins encoded in the representative genomic region were then scanned with the stand-alone version of InterProScan v5.10-50.0 (Jones et al., 2014). Final annotation of the representative gene cluster was based on the InterProScan results and on the Prokka annotation. Finally, the representative T3SS genomic cluster was used to perform a MultiGeneBlast search amongst all available GenBank genome entries. Classification of the *Pseudovibrio* T3SS was based on phylogenetic relationships. Homologous proteins to YscV and YscU were recovered from KEGG orthology, following the phylogenetic reconstruction reported by Gazi et al. (2012). Proteins were aligned using MUSCLE via the Guidance program v2.0 (Penn et al., 2010), and sequences with low confidence score were removed. The best evolutionary model was estimated using Prottest v3.4 (Darriba et al., 2011) allowing a GAMMA distribution, and maximum likelihood phylogenetic trees were reconstructed using RAxML, with 100 bootstrap repetitions.

### Prediction of the T3SS effectors

A *de-novo* prediction of the effectors was performed using EffectiveT3 v1.0.1, BPBAac, and T3_MM (Arnold et al., 2009; Wang et al., 2011, 2013). In order to minimize the number of false positives, we considered only the proteins predicted by all three tools. To this dataset were added proteins containing characteristic Pfam domains described for known T3SS effectors (Table S8; Arnold et al., 2010) and unannotated proteins encoded inside the *Pseudovibrio* T3SS gene clusters. The resulting list was used as query against the cd-hit protein-clusters. Clusters containing the query proteins were considered, and the amino acidic sequences of all proteins in the clusters were retrieved. This dataset was then filtered removing all proteins having a SEC-signal peptide, identified using the sensitive parameters in the SignalP server 4.1 (Petersen et al., 2011), and presenting a transmembrane domain in two out of three predictive tools used: TMHMM 2.0 server (Sonnhammer et al., 1998), HMMTOP 2.0 server (Tusnády and Simon, 2001), Phobius server (Käll et al., 2007). The filtered dataset was then clustered again using h-cd-hit as described above, and representative sequences were submitted to InterProScan. Results were manually screened and proteins containing domains unrelated to potential effectors were removed. Representative proteins were then submitted to the Phyre2 (Kelley et al., 2015), in order to infer potential functions based on structural homology with proteins structurally characterized. Finally, potential effectors were manually grouped according with their sequence similarity as detected via h-cd-hit, their domain architecture identified by InterPro, and their structural prediction obtained with Phyre2. Protein groups were then converted in a presence/absence matrix, used to perform a cluster analysis using Jaccard distances and the average clustering method in R.

### Annotation of the type IV and type V secretion systems (T4SS, T5SS)

Orthologous protein sequences for the T4SS structural components (VirB1-11, VirD4) were recovered from KEGG, and used to create HMM models which were then used to scan (*hmmscan*) the 12 *Pseudovibrio* proteomes, selecting a domain *E*-value of ≤ 10^−3^. Additionally, proteins containing the PF04610 (Trbl/VirB6) and PF04335 (VirB8) were recovered from all 12 *Pseudovibrio* proteomes, and integrated with the hits of the previous search. A representative genomic region containing adjacent genes potentially encoding component of the T4SS was then identified in the genome of strain W64. The contigs of the genome of this strain were then oriented using CONTIGuator v2.6 (Galardini et al., 2011), and the genome of *Pseudovibrio* FO-BEG1 (Bondarev et al., 2013) as reference. The representative genomic region encoding T4SS components was then selected in the oriented genome and used as query to look for homologous region in the 12 *Pseudovibrio* genomes using MultiGeneBlast. The proteins encoded in the identified gene cluster of strain W64 (PsW64_00918–00938) were then screened with InterProScan, and the results, together with the Prokka prediction, were used for gene annotation. Finally, the representative T4SS gene cluster in the oriented W64 genome was used as query in a MultiGeneBlast search against all available GenBank genome entries. Classification of the *Pseudovibrio* T4SS was based on the phylogenetic relationships of the VirB4 proteins. Proteins homologous to VirB4 were recovered from the Secret4 v1.0 database (Bi et al., 2013), aligned using MUSCLE via the Guidance program. Low confidence score sequences were not removed, because belonging all to the Type-I T4SS. Phylogenetic reconstruction was performed as described for the T3SS.

Elements of the T5SS were identified by selecting proteins containing the Pfam domains PF05860 (Haemagglutination activity domain), PF08479 (POTRA domain, ShlB-type), PF03865 (Hemolysin secretion/activation protein ShlB/FhaC/HecB), PF03797 (Autotransporter beta-domain). The genomic context of the positive hits belonging to the two-partner secretion (TPS) system was then inspected, in order to verify that the two components laid as adjacent genes and to discover potentially unidentified components. The obtained proteins were then used as query against the cd-hit protein-clusters. Representative sequences were recovered and scanned with InterProScan. Cd-hit protein-clusters were kept only if the domain architecture of the representative sequences was consistent with a T5SS component, and the resulting clusters were then manually grouped considering their domain architecture.

### Annotation of the type VI secretion system (T6SS)

The COGs characteristics for the T6SS (Boyer et al., 2009) were recovered for all 12 *Pseudovibrio* proteomes. Two representative genomic regions (T6SS-I: FO-BEG1_01844–01855; T6SS-II: FO-BEG1_02827-02846) containing adjacent genes encoding for the T6SS core components were selected, and used as query to look for homologous regions in the 12 *Pseudovibrio* genomes using MultiGeneBlast. Proteins encoded in the representative genomic region were then scanned with InterProScan. Gene annotation was based on the InterPro results and on the COG classification of the proteins. Finally, the representative T6SS gene clusters were used as query to perform a MultiGeneBlast search amongst all available GenBank genome entries. Classification of the *Pseudovibrio* T6SSs was based on phylogenetic relationships. Homologous protein to IglA and IglB were recovered from KEGG, following the phylogenetic reconstruction reported by Boyer et al. (2009). Phylogenetic reconstruction was performed as described for the T3SS.

### Prediction of T6SS effectors

VgrG-like proteins were retrieved from the 12 *Pseudovibrio* proteomes using their COG Ids COG3501. Sequences were screened with InterProScan and only proteins belonging to the RhsGE-associated Vgr family subset (IPR017847) or containing the Gp5 N-terminal (IPR006531) domain were used for further analysis. These sequences were then combined with an experimentally verified set of VgrG sequences recovered from the Secret6 database v2.0 (Li et al., 2015). Sequences were aligned using MUSCLE, best evolutionary model was estimated using Prottest, and a phylogenetic reconstruction was performed using RAxML allowing 100 bootstrap repetitions. Finally, for the phylogenetic clusters containing the proteins encoded by the “orphan” *vgrG* genes, a representative sequence was chosen and the genomic region in which it was present (FO-BEG1_01190–01198) was used as query in a MultiGeneBlast search against the 12 *Pseudovibrio* genomes, with the aim of recover homologous regions and inspect their gene composition.

In order to identify potential effectors belonging to the Tae and Tge group (Russell et al., 2014), we applied two independent approaches. As a first approach, a representative sequence of the experimentally verified effectors of each category was submitted to Phyre2 (Tae1: NP_250535.1; Tae2: ABC38716.1; Tae3: AAO68195.1; Tae4: CBA22870.1; Tge1: NP_252174.1; Tge2: NP_454891.1; Tge3: YP_109165.1). Proteins with confidence ≥ 90% were recovered and HMM models were built and used to perform a *hmmscan* of the 12 *Pseudovibrio* proteomes. In an independent search, the above approach was employed, but the initial HMM models were built using previously described effector and immunity proteins (Russell et al., 2012; Whitney et al., 2013). The two datasets were then combined. The same procedure was used for identifying proteins of the Tle group, creating HMM model using the sequences reported by (Russell et al., 2013). The obtained protein hits for all above classes of effectors were then used as query against the cd-hit protein-clusters, and the clusters containing the query proteins were recovered. For the effector proteins, sequences presenting a transmembrane domain and a Sec-signal peptide, identified as above, were removed. For each protein the respective genomic context and the structure homology predicted by Phyre2 were analyzed. Protein were considered as potential effectors only if they presented a smaller protein-coding gene encoding for a potential immunity protein in their close proximity, and if the Phyre2 structural homology search returned as first hit protein having a peptidoglycan hydrolytic activity, for Tae and Tge, or a lipase activity for Tle. The presence of the two characteristic catalytic motifs for the classes Tle1-4 (GxSxG) and Tle5 (HxKxxxxD) (Russell et al., 2013) was then assessed using FIMO v4.10.1 in the MEME suit (Grant et al., 2011). Effectors of the Tde group were identified recovering proteins containing domain PF15604 (Ma et al., 2014). This dataset was not further filtered because only one protein was identified. Finally, the isoelectric point of each protein hit was calculated using the ExPASy service (ExPASy, Compute pI/Mw tool^3^), and the SecretomeP 2.0 server (Bendtsen et al., 2005) was used to identify not canonical secreted proteins.

### Annotation of toxin-like proteins

The Pfam database was interrogated using the keyword “*bacterial toxin*,” and results were manually inspected to recover domains described to be present in known bacterial toxins. Additionally, we integrated this list with TIGRFAM domains present in toxin-antitoxin systems, and the domain TIGR03660, which was previously identified in *Pseudovibrio* toxin-like proteins (Romano et al., 2015; Table S9). Proteins containing the selected domains were retrieved and they were combined with the protein annotated by Prokka as toxin, and used as query against the cd-hit protein-clusters. Clusters containing the query proteins were recovered. The representative sequences of each protein-cluster were then scanned with InterProScan. If the representative sequences contained domains not directly attributable to bacterial toxin, the whole cluster was discarded. Cd-hit protein-clusters were then grouped according with the domain architecture of the representative proteins, and converted in an abundance matrix used to perform a cluster analysis as described above.

Potential toxin containing Rhs repeats were recovered from the Prokka annotation and from selecting proteins containing the domain PF05593 (Rhs repeat), and TIGR0164 (YD repeat 2 copies) and TIGR03696 (Rhs repeat-associated core domain). Positive hits were used as query against the cd-hit protein-clusters. Clusters containing the query proteins were recovered and their amino acidic sequences were annotated using InterProScan. Potential Rhs-toxins were then additionally submitted to Phyre2. Results were manually inspected and proteins having an InterPro annotation or Phyre2 prediction inconsistent with known Rhs-containing toxin were discarded. Potential immunity proteins Smi1/Knr4 were retrieved considering the Prokka annotation and the domain PF09346. Presence of potential toxin adjacent to the identified immunity protein was manually verified.

## Results

### General characteristics of the genomes, core-genome analysis, and phylogenetic inference

The genome size of the newly sequenced strains ranged from 5.1 Mbp of strain AD2 to 6.2 Mbp of strain AD14 (Table 1). The G+C content was quite consistent amongst the strains, ranging from 49.8 to 52.2%, with the only exception being the genome of strain AD26 that showed a G+C content of 45.2%. In general, the number of protein-coding genes was >5000 for all genomes; with the only exception being the genome of strain AD2, which had 4634 protein-coding genes. As all genomes were in draft format we assessed their quality on the basis of the presence of a set of 529 characteristic marker genes uniquely present in the family *Rhodobacteraceae*. This analysis showed that all genomes were of a high quality, with an estimated completeness of ≥98.7% (Table 1).

**Table 1:** **General features of the genomes used in the comparative analysis performed in this study**.

**Strain**	**Size (bp)**	**GC [%]**	**Fragments^b^**	**Gene number**	**Protein-coding genes**	**% Core-genome^a^**	**Completeness^c^(%)**	**GenBank Id**
AD13	6001312	50.64	36	5534	5468	57.9	99.7	LMCC00000000
AD14	6201736	50.02	57	5770	5679	55.7	99.7	LMCD00000000
AD2	5126200	50.3	162	4693	4634	68.3	98.7	LMCB00000000
AD26	6181400	45.17	159	5743	5680	55.7	99.4	LMCE00000000
AD37	5875058	50.0	224	5503	5445	58.1	99.4	LMCF00000000
AD46	6124061	49.79	85	5567	5502	57.5	98.7	LMCG00000000
AD5	6061014	49.87	66	5597	5497	57.6	99.4	LMCH00000000
FO-BEG1	5916782	52.5	2	5424 (5042; 382)	5309 (4927; 382)	59.6	99.7	PRJNA73563
JE062	5717078	52.15	53	5239	5096	62.1	99.0	PRJNA32093
W64 (AD8)	5935921	50.13	49	5458	5402	58.6	99.7	LMCI00000000
W74 (AD15)	6190724	50.59	64	5741	5686	55.6	99.6	LMCJ00000000
WM33 (AD30)	5745729	51.02	159	5394	5344	59.2	99.4	LMCK00000000

For the complete genome of FO-BEG1 the number of genes and protein-coding genes present in the chromosome and in the plasmid are reported in brackets.

a Proportion of protein-coding genes belonging to the consensus core-genome.

b Number of contigs or scaffolds.

c Completeness estimated considering single copy marker genes characteristic for the family Rhodobacteraceae.

In order to identify the core-genome, defined as the total number of orthologous protein families shared amongst all strains, we combined the orthologous protein family predicted by the COG, BDBH, and OrthoMCL algorithms, as implemented in GET_HOMOLOGUES (Contreras-Moreira and Vinuesa, 2013). The consensus core-genome resulted in 3164 shared families, representing in average 58.8 ± 3.5% of the protein-coding genes predicted in each genome.

**Figure 1:**
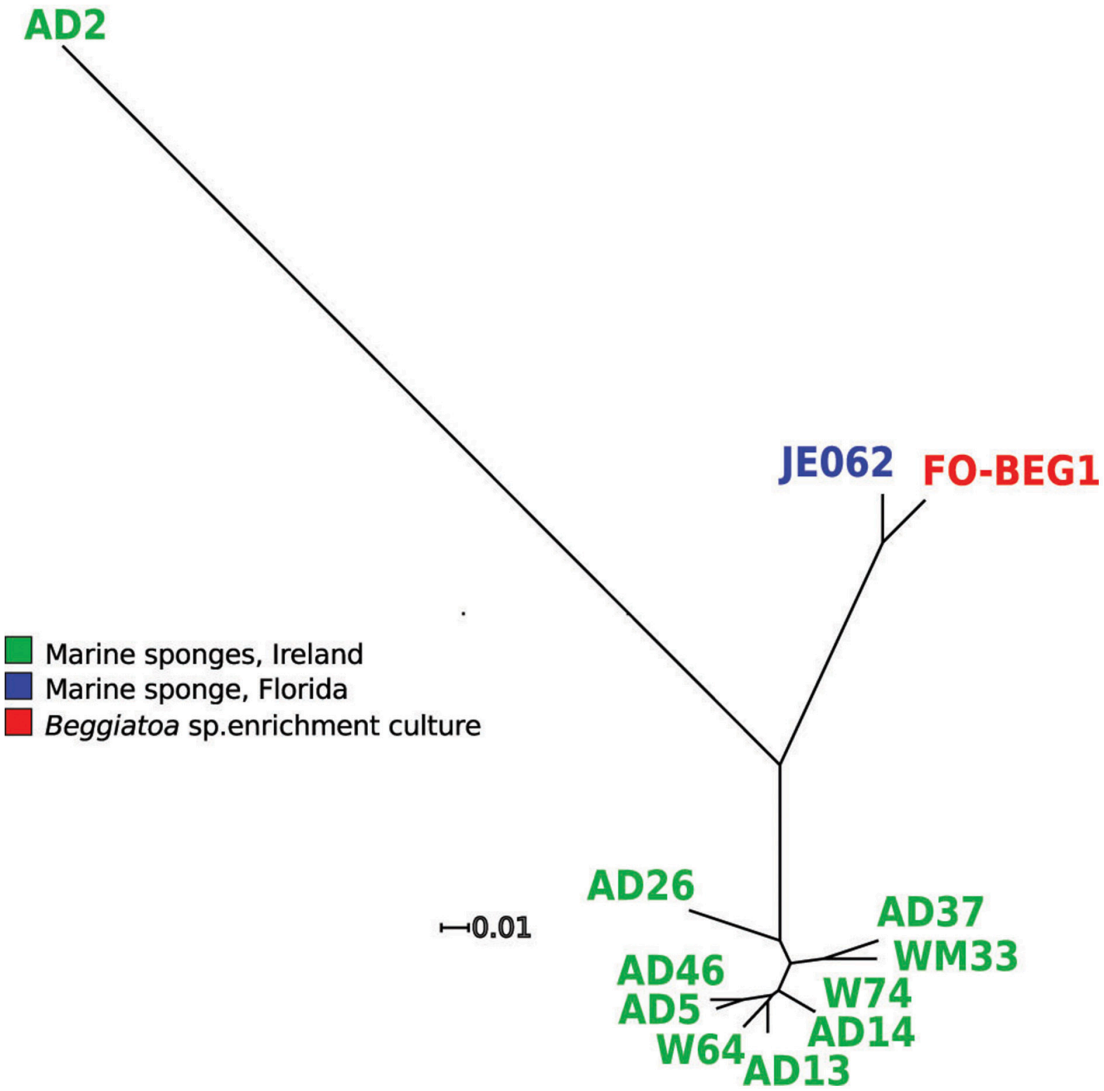
**Core genome phylogenetic tree**. The orthologous genes of the consensus core-genomes (3164) were used to perform a phylogenetic analysis, using the maximum likelihood algorithm (RaxML) and 1000 bootstrap repetitions. For all branches a support bootstrap value of 100 was obtained. The colors indicate the isolation source of the strains. Strain FO-BEG1 was isolated from an enrichment culture of *Beggiatoa* sp. strain 35Flor, which was originally obtained from a black band diseased scleractinian coral off the coast of Florida (Bondarev et al., 2013).

The genes coding for the proteins forming the core-genome were then used to perform a phylogenetic reconstruction (Figure 1). The most divergent branch in the phylogenetic tree was *P. axinellae* strain AD2, which formed an independent branch also in the phylogenetic tree constructed using the 16S rRNA gene sequence (Figure S1). A second distinct branch in the core genome tree was formed by strains FO-BEG1 and JE062, both related to *P. denitrificans* on the basis of the 16S rRNA gene (Figure S1). These results were consistent with the values of Average Nucleotide Identity (ANI) and Tetra nucleotide usage correlation (Tetra; Table S1). Both lineages, strain AD2 on the one side and strains FO-BEG1 and JE062 on the other, showed values well below the threshold of species definition (Richter and Rosselló-Móra, 2009), when compared with the other strains. All the other Irish isolates clustered together with the type strain *P. ascidiaceicola* in the 16S rRNA gene tree (Figure S1), sharing with it >99.4% 16S identity. Consistently, they all grouped together in a distinct third phylogenetic clade in the core genome tree (Figure 1). In this third clade (Figure 1, Figure S1) three sub-clusters were identified, with strain AD26 on the one hand and strains AD37 and WM33 on the other, forming separated branches from the rest of the isolates. Compared with the other strains, AD26 showed ANI values below the 95% species definition threshold (Richter and Rosselló-Móra, 2009; Table S1). However, the Tetra values did not support separation of this strain as a different species from the other member of this clade. Similarly, for both strains AD37 and WM33 neither the ANI values nor the Tetra values, obtained by comparison with the other strains of the third phylogenetic clade, were considerably below the thresholds used for species definition (Table S1).

### General metabolic features predicted in the genomes of the *Pseudovibrio* strains

To gain an overview of the predicted physiological features of the strains, we assigned each protein to Cluster of Orthologous Groups (COG) and annotated them using the KEGG database as reference. In general, >85.5% of the proteins could be assigned to a COG, with strain AD26 having the highest number of unassigned proteins (14.2%). Consistent with the high number of shared protein-coding genes, the distribution of the proteins amongst the COG categories did not show any clear difference amongst the strains (Figure S2). The majority of the genes identified were assigned to the COG categories E (Amino acid transport and metabolism), G (Carbohydrate transport and metabolism), and K (Transcription; Figure S2).

On average 46.7% ± 1.5% of the predicted proteins were annotated using the KEGG database. In order to identify shared metabolic features amongst the strains, we compared the KEGG annotations independently performed for each genome. Comparing the identified KEGG modules, it emerged that the predicted physiological features amongst the strains are highly conserved (Table S2). For example, all strains showed complete modules for “pyruvate oxidation” (M00307), “citric cycle” (M0009), “denitrification” (M00529), “fatty acid biosynthesis” (M00082, M00083), “beta-oxidation, acylCoA synthesis” (M00086), together with a wide array of modules related to the synthesis and degradation of nucleotide and amino acids, a wide array of cofactors and vitamin biosynthetic modules (e.g., thiamine, riboflavin, and coenzyme A biosynthesis; M00127, M00125, M00120), and a large number of transporter systems, such as molybdate, iron(III), putrescine, phosphate, oligopeptide, zinc, and heme transport systems (M00189, M00190, M00300, M00222, M00439, M00242, M00259). The only marked difference was observed in strain AD2, which was the only strain for which the module for the “assimilatory nitrate reduction” (M00531) was identified. The unique ability to assimilate nitrate as a nitrogen source by strain AD2 was confirmed experimentally by growing all isolates in defined medium with KNO_3_ as the sole nitrogen source (Figure S3). AD2 was the only isolate capable of growing under these conditions. The same strain showed other peculiarities, such as the absence of KEGG modules for the degradation of galactose, D-galactonate, D-galacturonate, and D-glucuronate. The modules related to bacterial secretion systems also differed, with the type IV secretion system module (M00333) identified with one block missing in seven out of 12 strains (W64, W74, AD2, AD5, AD46, AD26, AD14), and a complete type VI module (M00334) identified in all strains. The same is true for the type III secretion system module (M00332), which was identified with one block missing in all strains with the only exception being AD2.

### Type III secretion system (T3SS) and distribution of potential effectors in the *Pseudovibrio* genus

**Figure 2:**
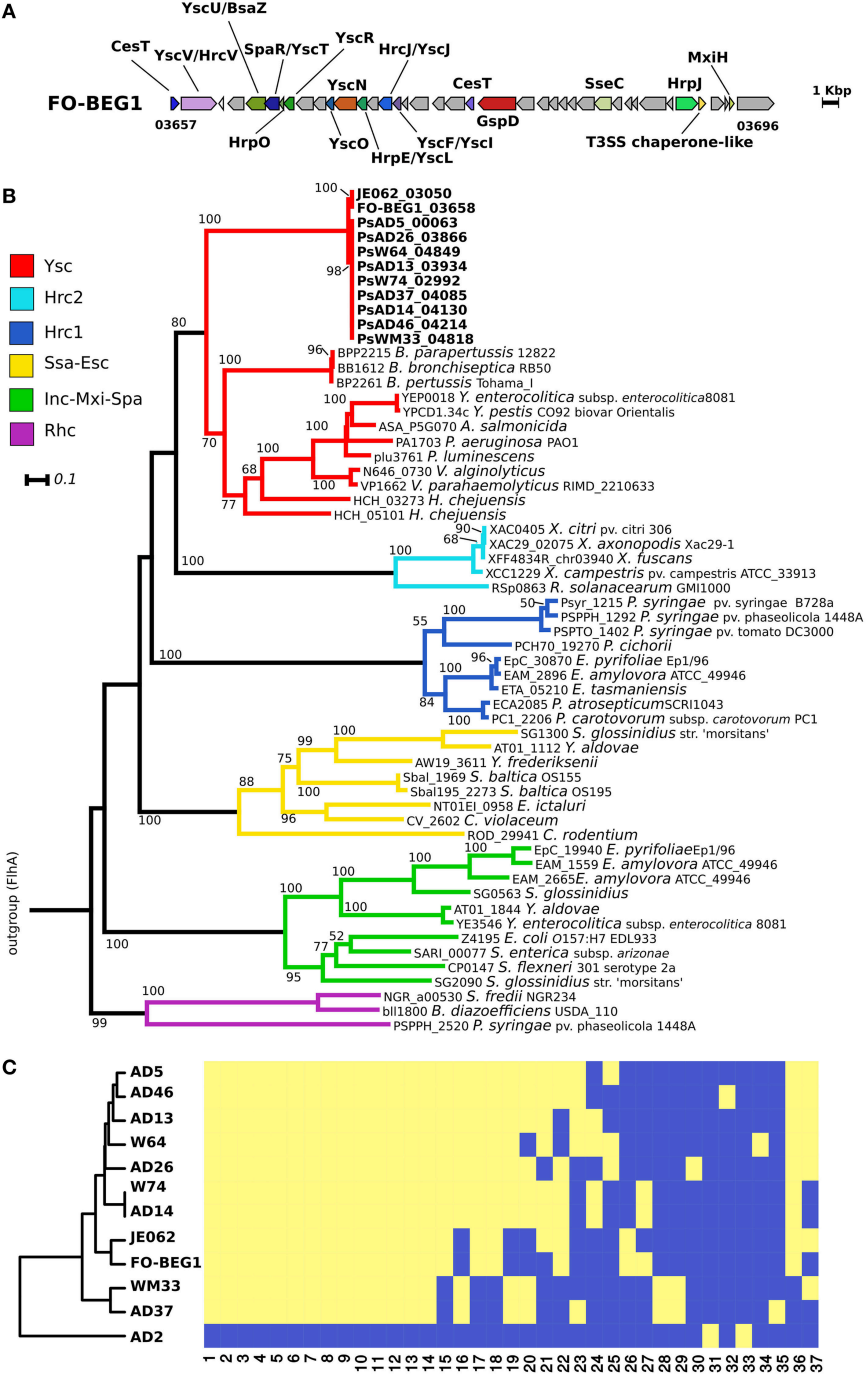
**Type 3 secretion system in the *Pseudovibrio* genus. (A)** Representative gene cluster coding for the T3SS identified in the *Pseudovibrio* genomes. All T3SS gene clusters identified in the 12 *Pseudovibrio* genomes are reported in Figure S4. The colors of the genes are consistent with those reported in Figure S4. Homologous genes shared amongst the 12 strains, but not identified as part of the T3SS structure with the approach we used, are colored gray. **(B)** Rooted phylogenetic tree reconstructed using proteins homologous to YscV. KEGG identifiers for each protein are reported. Only Bootstrap values higher than 50 are shown. Classification of the T3SS is based on the study of Gazi et al. (2012). Flagellar proteins FlhA were used as outgroup. **(C)** Cluster analysis performed using the presence/absence matrix obtained for the T3SS effectors identified in the *Pseudovibrio* genomes. The yellow and blue color indicates respectively, the presence or the absence of each effector type in the investigated genomes. The numbers below the heat-map refer to the effector groups obtained after clustering the proteins according to their sequence similarity, domain composition, and predicted structure. Details about each group are reported in Table S3.

T3SSs are directly involved in both pathogenicity and symbiosis, being essential mediators in the interaction between Gram-negative bacteria and eukaryotes via the direct injection of effector proteins inside the host cells (Dale and Moran, 2006; Preston, 2007). With the exception of strain AD2, we identified in all genomes a region coding for a T3SS (Figure 2, Figure S4A). The identified proteins were mapped on the KEGG module for the T3SS, revealing that all essential components were detected. The genomic organization of this cluster was highly conserved amongst all genomes. The only differences were observed in the genomes of strains WM33, AD13, and W74 (Figure 2A, Figure S4A). The T3SS gene cluster in *Pseudovibrio* had an unusual gene architecture, as underlined by the scarce homology in gene composition and organization shared with a gene cluster of *Labrenzia aggregata* strain IAM 12614, which resulted the most closely related cluster according with the MultigeneBlast search we performed (Figure S4B). Consistently, the phylogenetic analysis revealed that the T3SS proteins of *Pseudovibrio* form a well-defined, independent branch in the Ysc group of T3SS. This divergence was observed in both phylogenetic reconstructions performed using sequences of proteins homologous to YscV and YscU (Figure 2B, Figure S5).

We combined different prediction tools to identify potential T3SS effector proteins in the *Pseudovibrio* genomes (see Materials and Methods). This resulted in a wide array of positive hits that after being grouped according to their sequence similarity, domain architecture, and structural prediction resulted in 37 effector-groups (Figure 2C; Table S3). These data were then converted to a presence/absence matrix, which was used to perform a cluster analysis to verify the similarity of the strains according to the profiles of their putative T3SS effectors (Figure 2C). The robustness of the prediction approach we employed is underlined by the fact that only two potential effectors were identified in the genome of AD2, which lacks a T3SS. The clustering of the strains partially followed their YscV phylogenetic relationships, for example, following the phylogeny, strains FO-BEG1, and JE062 formed an independent branch that was separated from the other strains. Differently from the phylogenetic trees, these two strains formed a unique cluster with eight out of ten strains isolated off the coast of Ireland. The other two strains, AD37 and WM33, were clearly separated from the rest of the isolates, showing characteristic T3SS effector patterns (Figure 2C).

### Distribution of type IV and type V secretion systems (T4SS, T5SS) in the *Pseudovibrio* genus

**Figure 3:**
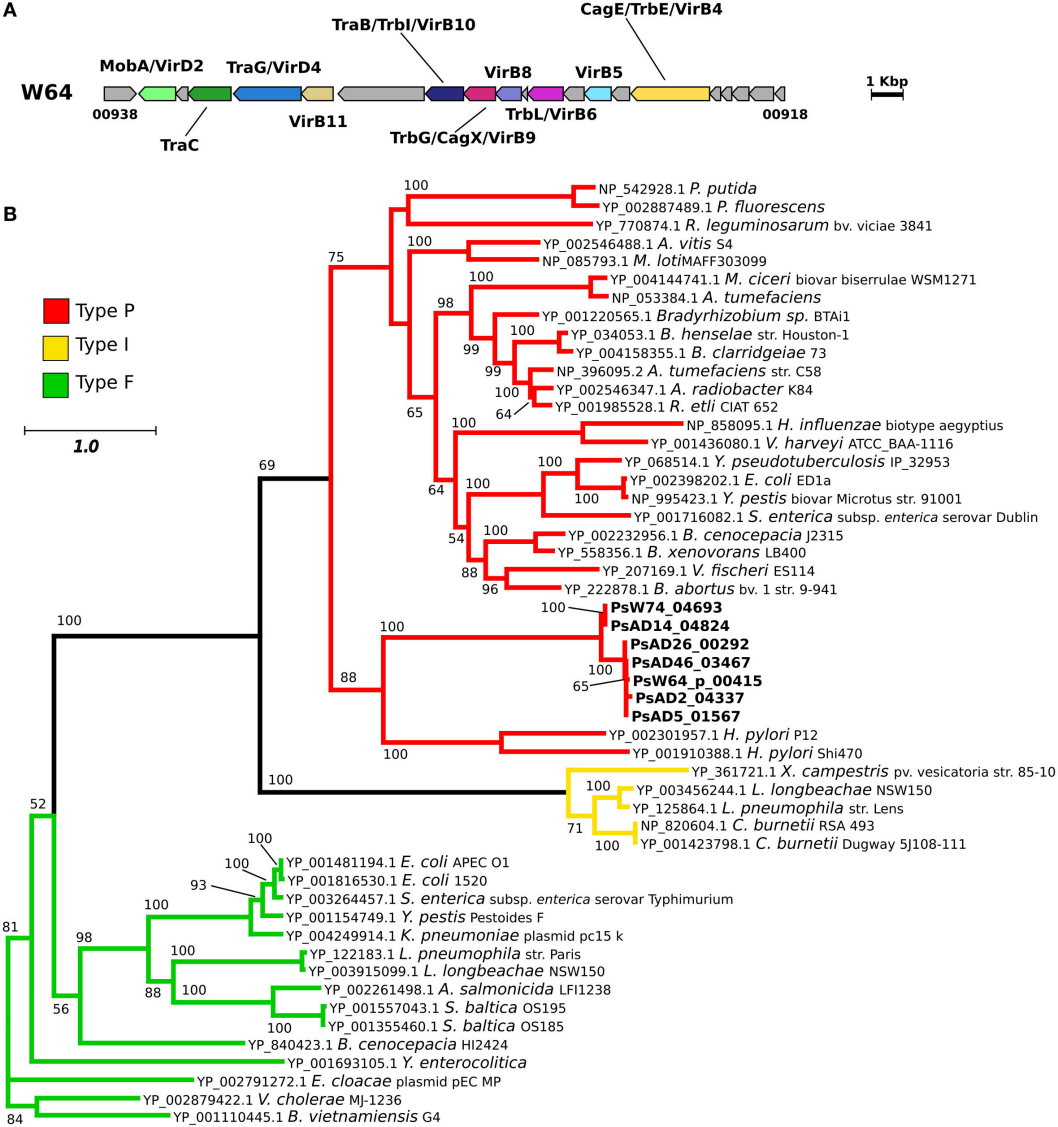
**Type 4 secretion system in the *Pseudovibrio* genus. (A)** Representative gene cluster coding for the T4SS identified in the *Pseudovibrio* genomes. All T4SS gene clusters identified in the 12 *Pseudovibrio* genomes are reported in Figure S6. The colors of the genes are consistent with the ones reported in Figure S6. Homologous genes shared amongst the 12 strains, but not identified as part of the T4SS structure with the approach we used, are colored gray. **(B)** Unrooted phylogenetic tree constructed using protein homologous to VirB4. NCBI accession numbers for each protein are reported. Only Bootstrap values higher than 50 are shown. Colors of the branches are based on the classification reported in the Secret4 database, following Lawley et al. (2003).

Similarly to T3SSs, T4SSs are cell envelope spanning complexes that deliver factors (DNA or proteins) from the cytoplasm of the donor to the recipient cells (Cascales and Christie, 2003). In agreement with the identified KEGG modules, we detected a conserved gene cluster potentially coding for a T4SS in seven genomes. This region was highly conserved in five out of seven strains (Figure 3A, Figure S6A), with the gene clusters identified in the genomes of strains AD14 and W74 showing the highest variability, being divided amongst multiple contigs, and having duplicated regions (Figure S6A). In the genome of strain AD14 the gene coding for the ATPase VirB4 was not detected in any of the genomic fragments containing T4SS components. Since this is a key protein conserved amongst the different types of T4SS, we manually scanned the genome of the strain to verify the presence of this gene. The gene encoding this protein was then identified in a contig containing this single ORF (Figure S6A). The T4SS gene cluster of the *Pseudovibrio* strains showed a high degree of similarity with clusters found in other members of the *Alphaproteobacteria*, such as *Labrenzia* sp. (Figure S6B). Finally, we classified the *Pseudovibrio* T4SS by performing a phylogenetic reconstruction using sequences of the well-conserved VirB4 protein (Figure 3B). This analysis revealed that the T4SS identified in the *Pseudovibrio* strains belong to the Type-P T4SS.

Differently from the T3SS and T4SS, T5SSs do not deliver proteins directly into the cytoplasm of a recipient cell, but they rather release proteins in the extracellular space (van Ulsen et al., 2014; Costa et al., 2015). The distribution and abundance of these systems in the genome of the *Pseudovibrio* strains was uneven, with no apparent link to their phylogenetic relationship (Table S4). With the exception of strain AD2, a variable number of proteins belonging to the T5SS containing autotransporter-like domain (IPR005546, IPR006315) and a pectin lyase fold/virulence factor (IPR011050) was identified in all strains (Table S4). The number of these proteins in each genome varied from 3, identified in strains FO-BEG1, WM33, W64 and AD37, to 7, identified in strain W74. Additional proteins containing autotransporter-like domains (IPR005546, IPR006315) or outer membrane protein beta-barrel (IPR027385), were identified in all strains, including a single occurrence in strain AD2 (PsAD2_00716; Table S4). Finally, components of the TPS were identified in seven strains, AD14, AD5, AD26, AD37, WM33, W74, AD46, with AD37 presenting two separate systems.

### Type VI secretion system (T6SS) and distribution of potential effectors in the *Pseudovibrio* genus

**Figure 4:**
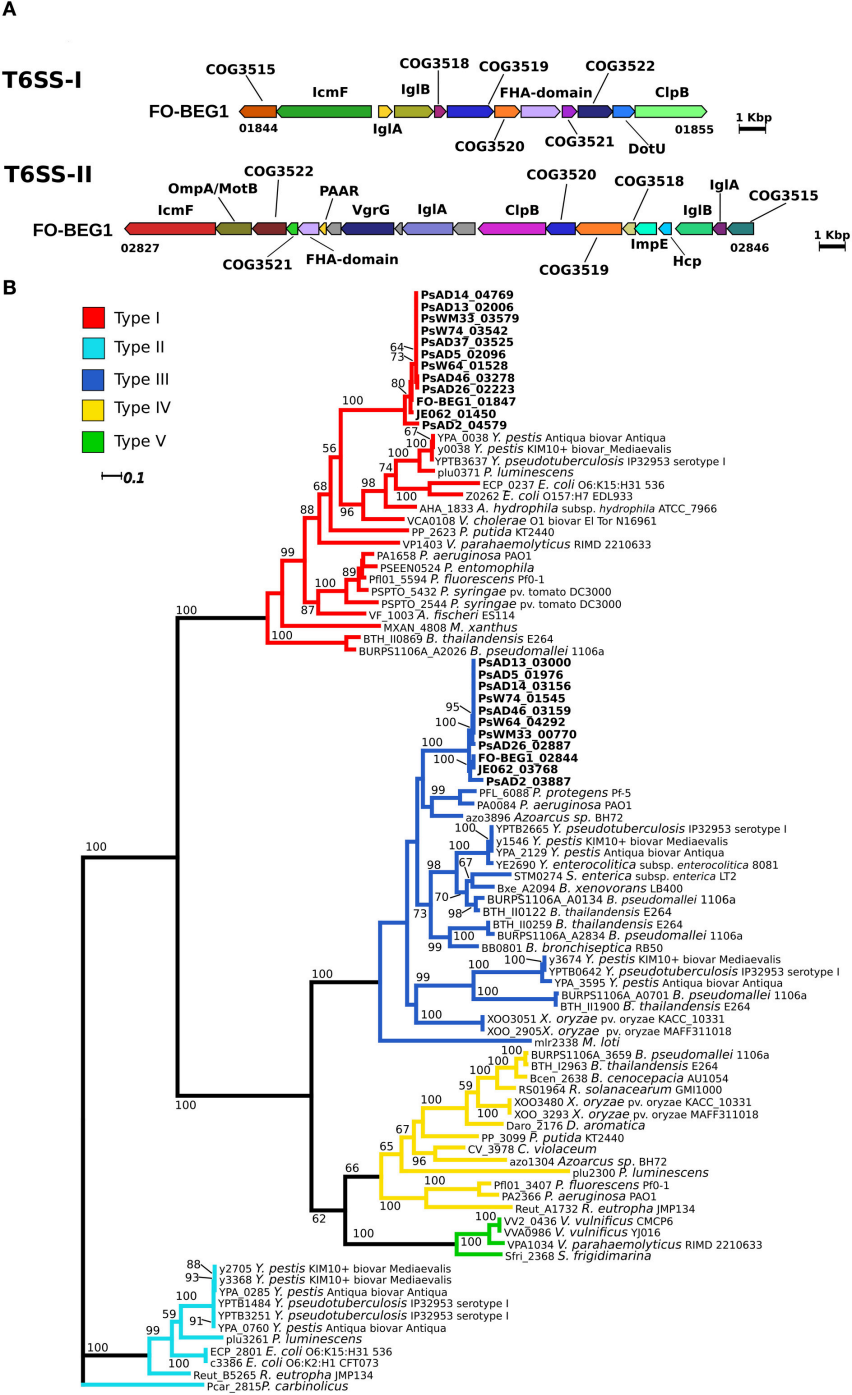
**Type 6 secretion system in the *Pseudovibrio* genus. (A)** Representative gene cluster coding for the T6SS identified in the *Pseudovibrio* genomes. All T6SS gene clusters identified in the *Pseudovibrio* genomes are reported in Figure S7. The colors of the genes are consistent with the one reported in Figure S7. Homologous genes shared amongst the 12 strains, but not identified as part of the T6SS structure with the approach we used, are colored gray. **(B)** Unrooted phylogenetic tree reconstructed using protein homologous to IglB. KEGG identifiers for each protein are reported. Only Bootstrap values higher than 50 are shown. Colors of the branches are based on the T6SS classification reported in Boyer et al. (2009).

**Figure 5:**
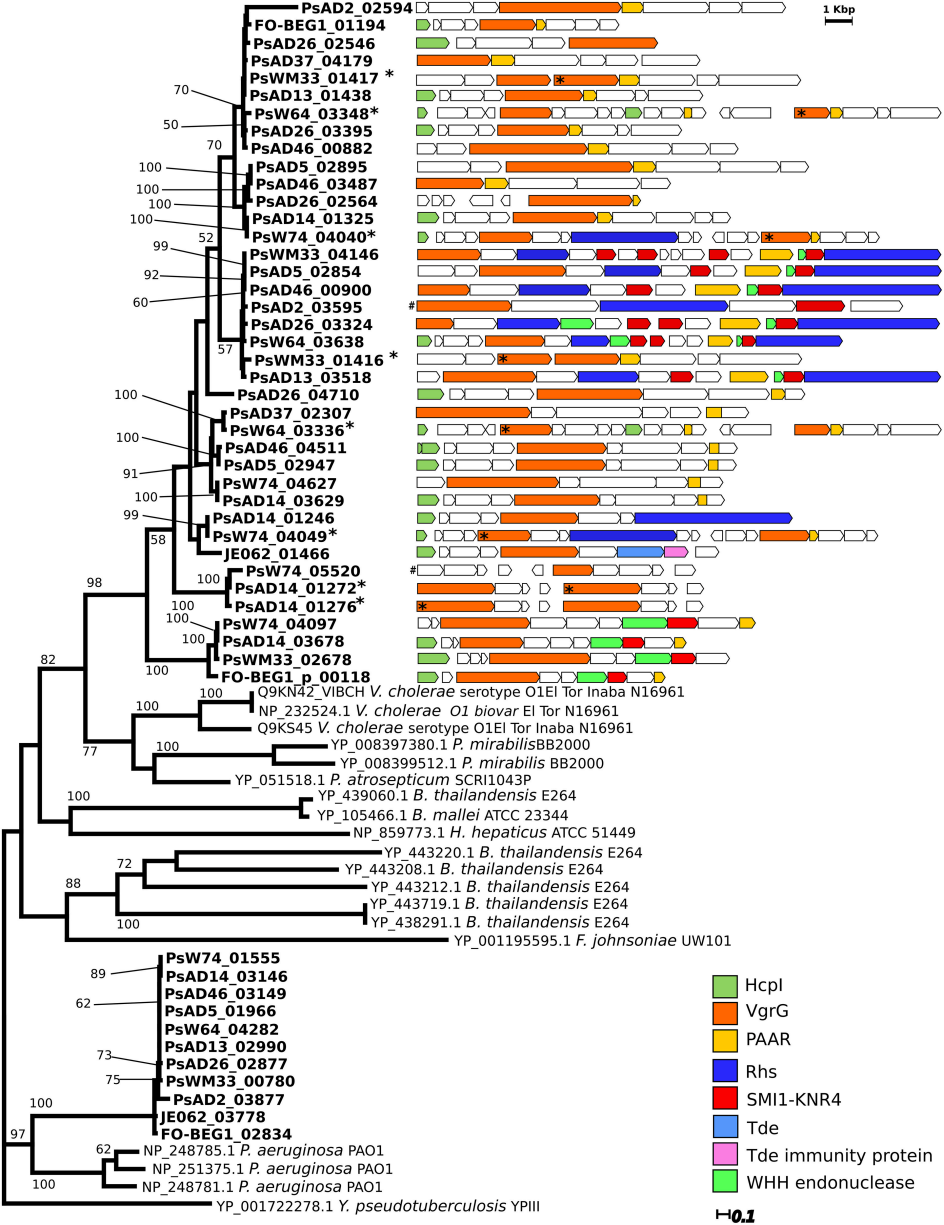
**Distribution of the gene coding VgrG-like proteins and comparison of their genomic context**. Experimentally verified VgrG protein sequences retrieved from the Secret6 database (Li et al., 2015) were included in the phylogenetic analysis. For these sequences NCBI or Uniprot accession numbers are reported. Only Bootstrap values higher than 50 are shown. The genomic organization and composition of the region containing the “orphan” *vgrG* genes were then analyzed performing a MultiGeneBlast search. Each genomic region is reported adjacent to the respective encoded VgrG protein present in the tree. Similar color indicates homologous genes of interest. In case the *vgrG* gene was present in duplicate in the genomic region, asterisks (^*^) associate the protein in the tree with the corresponding gene. The genomic region containing the genes PsAD2_03595 and PsW74_05520, indicated with a gate (^#^), were not retrieved from the MultiGeneBlast search. They were manually drawn and their dimension are not proportional to the other genomic regions in the figure.

The presence of a conserved gene clusters containing key components of a T6SS, but missing genes coding for the proteins VgrG and HcpI, was common to all strains (T6SS-I; Figure 4A; Figure S7A). The missing genes were identified in other genomic regions in all genomes (Figure 5). The *Pseudovibrio* T6SS-I gene cluster was highly similar to a cluster of the alphaproteobacterium *Roseibium* sp. strain TrichSKD4, which like *Pseudovibrio* belongs to the order *Rhodobacterales* (Figure S7B). A second gene cluster (T6SS-II) containing all 13 T6SS key components, according to Boyer et al. (2009), was identified in the genome of all *Pseudovibrio* strains, with the exception of AD37 (Figure 4A; Figure S7C). The genomic organization of these clusters was different from the T6SS-I, and it also contained the *hcpI* and *vgrG* genes. The T6SS-II cluster showed partial similarity with gene clusters belonging to the gammaproteobacteria *Methylomicrobium alcaliphilum* str. 20Z, and *Pseudomonas aeruginosa* strain PUPa3 (Figure S7D). Finally, we classified the T6SSs of *Pseudovibrio* performing a phylogenetic reconstruction using the IglA and IglB proteins and following the aforementioned Boyer classification and in both analyses the T6SS-I proteins clustered together with sequences belonging to the type I T6SS, whereas the proteins of the T6SS-II grouped together with sequences of the type III T6SS (Figure 4B, Figure S8).

We then looked for the presence and distribution of the more common known T6SS effector proteins amongst the 12 *Pseudovibrio* strains. Multiple VgrG-like proteins were identified in the 12 genomes, and peculiarly they formed two clearly separated clusters in the phylogenetic reconstruction we performed (Figure 5). All VgrG proteins identified inside the T6SS-II formed a defined clade, supported by robust bootstrap values, together with sequences belonging to *Pseudomonas* sp. (Figure 5). This was consistent with both the phylogenetic reconstruction performed using the structural components of the T6SS, and the gene cluster comparison with the available bacterial genomes (Figures 4A,B; Figure S5). The other clade contained multiple VgrG proteins encoded by genes which were not located within a T6SS gene cluster. These “orphan” *vgrG* genes were identified in all *Pseudovibrio* strains. Inspection of the genomic regions containing the “orphan” *vgrG* genes revealed a high degree of variability in both gene content and organization (Figure 5). Most of these genomic regions also contained the other essential gene, *hcpI*, missing from T6SS-I. The only exceptions were strains AD2, and AD37, for which the genes encoding the HcpI protein were located outside these regions (PsAD37_02594, PsAD2_03886, and PsAD2_04272).

Recently, different T6SS effector proteins targeting the bacterial cell wall (Tae, Tge), membrane (Tle), or nucleic acid (Tde) have been identified, alongside immunity proteins that protect the injecting cells from auto-intoxication (Russell et al., 2014). Effectors belonging to the category Tae and Tge share properties, such as being in a bicistronic operon with their immunity protein genes, having an isoelectric point (iP) >8, and not possessing a Sec-signal peptide and transmembrane domains (Russell et al., 2014). We screened the *Pseudovibrio* genomes in an attempt to identify potential effectors with these characteristics. Apart from FO-BEG1, JE062, and AD2, potential Tae-like effectors were identified in all *Pseudovibrio* genomes (Table S5). Even though we did not identify structural homology with characterized Tae proteins, additional analyses revealed that all potential effectors presented other features consistent with known Tae (shorter gene downstream potentially coding immunity proteins, iP >8, absence of Sec-signal peptide and transmembrane domain). Moreover, all potential effectors had structural homology with proteins involved in peptidoglycan degradation, as the known Tae. No significant structural homology with characterized Tae immunity proteins was obtained for the hypothetical immunity proteins encoded by the genes downstream the potential Tae effectors. Surprisingly, we identified Tae immunity proteins of the Tai4 family in FO-BEG1 and JE062. Consistent with our prediction, these shared a high structural homology to Tai proteins characterized in *Serratia* (Table S5). However, we could not identify genes encoding potential effector proteins in their genomic proximity.

Interestingly, in all but one genome (AD2) we identified potential Tle effectors. In general up to two potential effectors were identified in each genome, with the exception of strain AD26 which presented four Tle-like proteins. The structural homology with the Tle effectors previously described in *Pseudomonas* and the presence of the characteristic GxSxG motif, with the only exception being the proteins belonging to strain AD37, strongly suggest that all the identified Tle-like proteins might be T6SS effectors with lipase activity. However, a potential upstream immunity protein was present in only seven of the 17 potential Tle effectors (Table S5). These seven effectors had peculiar characteristics, having the GxSxG motif localized in a different region of the protein than the others, showing the lowest iP, and the highest likelihood of being secreted according to the SecretomeP prediction (Table S5). Altogether these data indicates that, despite the structural homology with known Tle's shown by all 17 effectors, the aforementioned seven proteins may represent the more likely candidates. Finally, confirming previous analyses (Ma et al., 2014) we could identify in strain JE062 a Tde effector (JE062_01464) and the respective immunity proteins (JE062_01465; Table S5), which were in close proximity to an “orphan” *vgrG* gene (Figure 5). Strikingly, none of the other *Pseudovibrio* genomes harbored genes encoding similar effectors.

### Annotation and distribution of potential toxin-encoding genes in the *Pseudovibrio* genus

**Figure 6:**
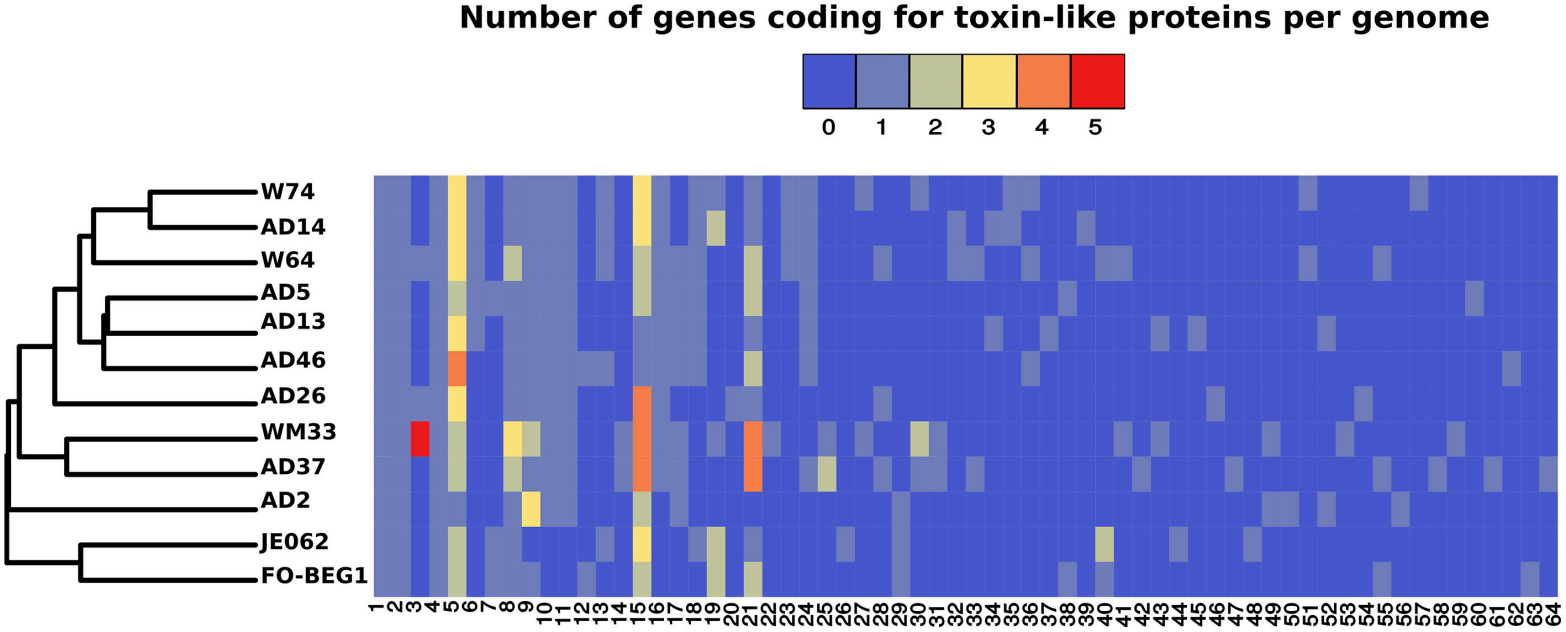
**Distribution of genes encoding toxin-like proteins in the *Pseudovibrio* genus**. The cluster analysis was performed using an abundance matrix constructed considering the distribution of the genes encoding toxin-like proteins amongst the 12 *Pseudovibrio* genomes. Colors in the heat-map indicate the number of the toxin-like genes belonging to each toxin-group. Toxin-groups were obtained after clustering the proteins potentially encoding toxins according with their sequences similarity and domain/repeat architecture. Each group is indicated as a number reported below the heat-map. Details about each toxin-group are reported in Table S6.

The approach we used to identify toxin-like proteins, based on gene annotation and a search for the presence of specific Pfam domains, resulted in a total of 303 positive hits. Marked differences in abundance were observed amongst the strains, with strain AD2 having only 18 toxin-coding genes while strains AD37 and WM33 had 34 and 40, respectively (Table S6). In all other strains we identified on average 23 ± 4 toxin-coding genes. The majority of these proteins contained a serralysin-like metalloprotease C-terminal (IPR011049), a hemolysin-type calcium binding-related domain (IPR010566), and hemolysin-type calcium-binding repeats (IPR001343; Table S6). We applied conservative criteria to group these proteins according to their sequence similarity and their domain/repeat architecture. The distribution of the toxin-groups we obtained were then converted into an abundance matrix and used to perform a cluster analysis. The resulting dendrogram partially followed the phylogenetic analysis we performed using the core-genome, emphasizing, however, the divergence of strains FO-BEG1, JE062, and AD2 on one side and strains WM33 and AD37 on the other (Figure 6). The same is true for strain AD26, which was well-separated from the other strains obtained from the Irish coast (Figure 6; Table S6).

While identifying components of the T6SS we observed that in proximity to the “orphan” *vgrG* genes annotated as Smi1/Knr4 and as Rhs-containing proteins were often present. Recently, it has been shown that these two proteins might represent a new class of toxin-immunity protein pair, and in some cases the Rhs-containing proteins have been described as T6SS effectors (Zhang et al., 2011; Russell et al., 2014). In order to investigate the distribution of this system in the *Pseudovibrio* genomes we examined all genes encoding both the potential immunity and the toxic proteins. With the exception of strain JE062, this system was identified in all *Pseudovibrio* genomes (Table S7). In almost every strain the genes coding for this toxin-immunity system were located in close proximity to each other and to the “orphan” *vgrG* genes (Figure 5; Table S7). The highest number of genes encoding Smi1/Knr4-like proteins were identified in strains AD2 and AD37, and peculiarly in both strains these regions were not in proximity to the “orphan” *vgrG* genes. It is important to mention that for 11 out of 12 strains only draft genomes are available, therefore the differences we have observed in the genomic organization of these toxin-immunity islands amongst the strains may in fact be due to sequencing gaps. Smi1/Knr4-like immunity proteins have been described to protect the producing cells from a wide array of nucleic acid degrading toxins (Zhang et al., 2011) including toxins containing the Pfam domain PF14414, characteristic of WHH nucleases belonging to the HNH/ENDO VII superfamily. Interestingly, we identified genes potentially encoding proteins containing such domain in proximity of the Smi1/Knr4-like immunity proteins in almost all *Pseudovibrio* strains, with the exception of strains W74 and JE062 (Table S6, Figure 5). Moreover, in the majority of the strains these potential toxins were located close to genes coding for the Rhs-containing proteins in the genomic region containing the “orphan” *vgrG* genes (Figure 5; Table S7).

## Discussion

### Strains of the *Pseudovibrio* genus share a high degree of genetic and metabolic features

Overall the general genomic features, the phylogenetic analyses, and the ANI and Tetra values showed a high degree of similarity amongst the investigated strains, with *P. axinellae* strain AD2 being the most divergent lineage (Figure 1A, Table 1, and Table S1). The 12 strains share a high portion of their genomic content, with almost 60% of each strain's protein-encoding genes being part of the consensus core-genome (Table 1). The dimension of the core-genome can vary greatly depending on the taxonomic group used for the comparative genomic analysis (e.g., species, genus, family) and on the number of genomes considered. Previous studies conducted at the species and genus level for other taxonomic groups indicate that the core-genome can represent up to 40% of the genome of each strain (Welch et al., 2002; Lefébure and Stanhope, 2007; Seo et al., 2015). Altogether, these data underline the homogeneity in terms of gene content amongst the *Pseudovibrio* strains.

The high metabolic versatility that emerged from the KEGG and the COG annotations (Figure S2; Table S2), points toward a generalist lifestyle that allows *Pseudovibrio* to thrive under a wide variety of environmental conditions. This is consistent with previous reports that have identified *Pseudovibrio* strains in different ecological contexts (Bondarev et al., 2013). Notably, a KEGG module involved in assimilatory nitrate reduction was identified only in the genome of strain AD2. The functionality and uniqueness of this system to the AD2 strain amongst all the *Pseudovibrio* isolates was confirmed in KNO_3_-supplemented growth assays (Figure S3). To the best of our knowledge this metabolism has never been described in the *Pseudovibrio* genus. In marine sponges, the most common host of *Pseudovibrio*, experimental data has shown a high rate of nitrification, the biological conversion of ammonia to nitrate via nitrite. It is likely therefore that, at least under certain conditions, nitrate may be used as a nitrogen source by part of the sponge microbiota (Taylor et al., 2007). Key enzymes involved in assimilatory nitrate reduction have also been identified in the sponge-specific lineage *Candidatus* Poribacter, where it has been suggested that this pathway might be useful under conditions of seasonal ammonia limitation (Siegl et al., 2011). This suggests that, in contrast to the other *Pseudovibrio* strains, AD2 possesses a genomic signature pointing toward a specific adaptation to the environmental conditions found in the sponge host.

### Mechanisms potentially used by *Pseudovibrio* to interact with the host and its microbiota

Considering the homogeneity in terms of gene content and predicted physiological features, and considering that the majority of the investigated strains were isolated from the same location and same type of sponge off the coast of Ireland, we decided to investigate the distribution of systems that are known to play an important role in prokaryote-prokaryote and prokaryote-eukaryote interactions (Dale and Moran, 2006; Kapitein and Mogk, 2014; Costa et al., 2015). These systems could influence the lifestyle (e.g., mechanisms used to colonize the host, mechanisms for interacting with the host microbiota) of the *Pseudovibrio* strains, allowing them to coexist in the same bacterial community. Sequencing of the *Pseudovibrio* genomes revealed a large cohort of secretion systems, each of which has been described in a broad spectrum of symbiotic (whether pathogenic, mutualistic, or commensal) interactions. Although the role of each of these secretion systems in the interaction between bacteria and sponges remains to be elucidated, studies of homologous systems in other hosts suggests that they will be key to the bi-directional signaling dynamics between microbe and host (Woolridge, 2009; Costa et al., 2015).

While each of the *Pseudovibrio* isolates investigated in this study possesses secretion potential, the dynamics of production and the nature of the secretome can vary between strains. Phylogenetic reconstruction of the T3SS of *Pseudovibrio* indicates that it belongs to the Ysc family, named after the archetypal T3SS of *Yersinia* spp (Figure 2, Figure S5; Cornelis, 2002). This relationship, together with the presence of several families of T3SS effectors, including IpgD/SopB, OspF/SpvC, YopJ, and YpkA-like, suggest that *Pseudovibrio* might use this system in a similar way to other well-known pathogenic bacteria. In these organisms the T3SSs are mainly used to reduce phagocytosis and block the mitogen-activated protein (MAP)-kinase and consequently the inflammatory response of the host (Norris et al., 1998; Cornelis, 2002; Niebuhr et al., 2002). Consistently, in Cnidaria and Porifera, the two more common potential hosts of *Pseudovibrio*, an innate immune response system that relies on the presence of MAP-kinase signaling cascade for its activation has been described (Müller and Müller, 2003; Miller et al., 2007). The distribution of effector proteins was not uniform amongst the *Pseudovibrio* isolates. For example, strains FO-BEG1, AD14, and W74 were the only strains that had a YopJ-like effector, while the YpkA-like effector was identified in all but four strains (AD26, AD37, WM33, FO-BEG1; protein-group 21 in Figure 2C and Table S3). Interestingly, although strain AD37 clustered together with strain WM33, we could not identify in the former any potential effectors that have the potential to interfere with the host inflammatory response (e.g., YpkA-like, YopJ, OspF/SpvC; Table S3). Overall, these data underline the diversity of mechanisms that *Pseudovibrio* strains can employ to interact with the host cell, suggesting that phylogenetically and metabolically related bacteria adopt different colonization strategies to thrive and co-exist within the host. Moreover, the absence of a T3SS in strain AD2 further highlights the divergence of this strain from the others, indicating that AD2 is either unable to penetrate into the host cell or adopts different mechanisms to avoid digestion during infection.

The distribution of the T4SS did not follow the phylogenetic relationships of the strains (Figure 3, Figure S6), perhaps a reflection of the frequency with which these elements are horizontally acquired (Cascales and Christie, 2003). Using the classification reported in Lawley et al. (2003), phylogenetic reconstruction showed that the *Pseudovibrio* strains possess a Type-P T4SS (Figure 3). The Type-P T4SS appears to be able to transfer/secrete/take up a wide repertoire of macromolecules (Lawley et al., 2003), including proteins and DNA. The presence in the *Pseudovibrio* T4SS gene clusters of genes encoding for proteins containing domains involved in nucleic acid manipulation (e.g., *Toprim_3*, PF13362, in PsW64_00935), and for a relaxase/mobilization protein (MobA/VirD2; PsW64_00937; Figure 3A, Figure S6) that plays an essential role in the conjugative DNA transfer (Byrd and Matson, 1997), suggests that the T4SS of *Pseudovibrio* may be mainly involved in mobilizing DNA. Finding this secretion system in *Pseudovibrio* is noteworthy, since the release and uptake of DNA may contribute to gene exchange and genome plasticity allowing the strains to adapt to changing environments. However, a role in the delivery of effector proteins cannot be excluded since it has been described that the T4SS of *Agrobacterium* is both a conjugation system and an injector of protein effectors (Cascales and Christie, 2003; Bhatty et al., 2013).

With the exception of strain AD37, two gene clusters coding for a T6SS were identified in all *Pseudovibrio* strains. To date the two main functions described for the T6SS are the delivery of protein effectors into host cells and the antagonistic role toward other bacteria (Russell et al., 2014). VgrG proteins are essential components of the T6SS and besides having a structural role, they are known to degrade peptidoglycan, cross-link host cell actin, and mediate membrane fusion during the intracellular spread of pathogenic bacteria (Russell et al., 2014). Most of these VgrG proteins present specific functional domains, which we could not identify in the *Pseudovibrio* proteins with the approaches we used. This suggests that the *Pseudovibrio* VgrG proteins may mainly play a structural role. T6SS systems are proposed to help bacteria to conquer an ecological niche (Kapitein and Mogk, 2014; Ma et al., 2014; Russell et al., 2014), and indeed niche specific distribution of T6SS effectors has recently been described (Egan et al., 2015). The *Pseudovibrio* genomes were found to encode potential effectors belonging to the Tae, Tle, and Tde categories (Table S5), although the distribution and abundance of these effectors is not entirely consistent with the phylogenetic relationship of the strains. The most obvious example is the presence of the Tde effector only in strain JE062. It has recently been reported that this type of effector can be used by *A. tumefaciens* to outcompete *P. aeruginosa in-planta*, providing the first evidence that T6SSs enable bacteria to settle in natural environments (Ma et al., 2014). As before, no potential effectors were identified in the genome of strain AD2, again suggesting a different colonization strategy adopted by this strain to establish itself within the host microbiota.

T5SSs have been described as having very different functions, such as hydrolysis of macromolecules and adhesions to host cells, and the best studied examples are the classical monomeric autotransporters, and the two-partner secretion (TPS; van Ulsen et al., 2014). The first consists of multi-domain proteins that after insertion in the outer membrane facilitate the translocation of passenger domains, which are responsible for the interaction with the environment. The second is formed by two proteins: a secreted protein containing functional domains, and an outer membrane transporter protein (van Ulsen et al., 2014). With the exception of AD2, in all *Pseudovibrio* strains monomeric autotransporters containing variants of the pectin lyase fold/virulence factor (Table S4) were identified. These domains include virulence factors involved in binding and degrading polysaccharides such as pectin and carrageenan (Jenkins et al., 1998), which are polymers found in the cell wall of plants and red-algae, respectively. Pectin lyase fold domains are also present in the adhesin autotransporter protein AIDA, which in diarrheagenic *E. coli* is responsible for adhesion of cells to a wide array of human and non-human cell types, and is involved in bacterial aggregation and biofilm formation (Sherlock et al., 2004). These systems may play a similar role in cell-to-cell or cell-to-host adhesion in *Pseudovibrio.* In fact it is worth considering that *Pseudovibrio* have been found associated with red-algae (Bondarev et al., 2013), and that such algal particles can be accumulated in the sponge mesohyl as a consequence of the filtration activity. Major differences were observed in the distribution of the TPS system, being identified in seven out of 12 strains (Table S4). Interestingly, all secreted proteins had a hemagglutinin-like domain or repeats, commonly found in proteins used for adhesion by the whooping cough agent *Bordetella pertussis* (Kajava et al., 2001; Hodak et al., 2006). Only in strain AD26 the secreted components of the TPS contained a bacterial EndoU nuclease domain, and in WM33 a second potential secreted component presented a VENN motif-containing domain (Table S4). Both these domains have previously been described in polymorphic bacterial toxin (Aoki et al., 2010; Zhang et al., 2011). Altogether, these similarities suggest that in general the TPS is used by *Pseudovibrio* for adhesion purposes, with the exception of strains AD26 and WM33, which might use it to deliver toxic proteins. It is important to stress out that the above described systems were not identified in the genome of strain AD2, underlying once more the divergence of strategies adopted by this strain to interact with the surrounding environment.

### Diversity of toxin-like proteins and new virulence islands identified in the *Pseudovibrio* genus

A large number of toxin-coding genes were identified in all 12 *Pseudovibrio* genomes (Tables S6, S7; Figure 6). Secretion of these toxins may occur either dependent or independent of some of the secretion systems described above. For example, some endonuclease Rhs-containing toxins can be transferred between bacterial cells in a manner that depends on the presence of adjacent VgrG-coding genes (Russell et al., 2014). Independently from the modality of secretion, Rhs-containing proteins can fulfill an antagonist role against both prokaryotes and eukaryotes (Kung et al., 2012; Russell et al., 2014). The Rhs proteins identified in the *Pseudovibrio* genomes were generally within the genomic region containing the “orphan” *vgrG* genes, and some of them shared structural homology with the Tc toxins of pathogenic bacteria (Figure 5; Table S7; Meusch et al., 2014). These data suggest that the *Pseudovibrio* Rhs-containing proteins could be delivered by T6SS and used against eukaryotes. Intriguingly, Rhs-containing toxin have been shown to be used by bacterial symbionts to protect aphids against parasitoids (Oliver et al., 2010), suggesting that *Pseudovibrio* may use this system as a weapon to protect the host. The genomic regions containing the “orphan” *vgrG* presented several proteins belonging to the SMI1/KNR4 family, which have been described as a new type of immunity proteins often associated with nuclease toxins containing Rhs or WHH domains (Zhang et al., 2011). Genes encoding proteins with WHH domains were also located in the same *vgrG* containing region in the *Pseudovibrio* genomes (Figure 5; Table S6, S7). These data indicate that these newly described systems are also present in *Pseudovibrio*, and that the genomic regions containing the “orphan” *vgrG* genes represent additional virulence islands previously unknown in this genus. The distribution and genomic organization of these putative islands is variable within the genus, perhaps reflecting the complexity of community dynamics within the host.

The majority of the toxin-like proteins we identified presented a serralysin-like metalloprotease (Table S6). Serralysin is a bacterial Zn-endopeptidase that has been widely studied in *Serratia* sp. They are considered important virulence factors, being able to generate tissue damage, degrade humoral proteins and tissue components involved in eukaryotic defense, and suppress *in-vitro* phagocytosis (Molla et al., 1986; Park and Ming, 2002; Ishii et al., 2014). Many of the toxin-like proteins identified in the *Pseudovibrio* genomes contained hemolysin-type calcium-binding repeats, which are nonapeptides characteristic of a family of proteins known as Repeats-in-toxin (Rtx; Linhartová et al., 2010). Most of these repeats are associated with metallopeptidase domains in the *Pseudovibrio* toxins, similarly to the above mentioned proteases from *Serratia.* In *Pseudomonas* it has been shown that a similar Rtx protease (AprT) cleaves fibrin, fibrinogen, and soluble laminin (Linhartová et al., 2010). Finding proteins with this domain composition in *Pseudovibrio* is noteworthy, as the extracellular matrix of sponges is rich in proteoglycans, lamin-like subunits, fibronectin and other structural proteins (Har-el and Tanzer, 1993; Ozbek et al., 2010), suggesting that such secreted proteins can help *Pseudovibrio* to penetrate into the sponge mesohyl.

Over the last number of years, it has become clear that the above described secretion systems and toxins are not exclusive to pathogens, and are also present in free-living and symbiotic bacteria (Dale and Moran, 2006; Persson et al., 2009). This is particularly true when symbionts are acquired from the surrounding environment, because they would need mechanisms to engage with the host, penetrate or adhere to the extracellular matrices, and eventually interact and/or infect the host cells avoiding digestion. It has also often been described that when symbiotic bacteria are vertically transmitted and have an ancient symbiotic relationship with their hosts, a progressive gene reduction occurs, with many of the systems used to overcome host defense being lost (Dale and Moran, 2006; McCutcheon and Moran, 2012). Compared to the other *Pseudovibrio* strains, strain AD2 was characterized by a reduction in genome size, secretion systems (e.g., T3SS, T5SS), their effectors and toxins (Figures 2, 6, Figure S4; Table 1, Tables S3–S6). Additionally, this strain formed a clear diverged branch in the phylogenetic analyses we performed (Figure 1, Figure S1). With this in mind it is tempting to speculate that strain AD2 might be a *Pseudovibrio* lineage having an ancient association with the host and which may be vertically transmitted to the progeny. Variability in the distribution and abundance of the secretion systems and toxins was also evident amongst the other *Pseudovibrio* strains, suggesting that they can use different mechanisms to interact with the eukaryotic hosts and its microbiota. This is particularly interesting given that nine out of the twelve strains analyzed in this study were isolated from the same sponge and geographical location (O'Halloran et al., 2011). Notwithstanding their similarity in terms of physiological potential (Figure 1, Figure S1, Table S2), the different mechanisms involved in prokaryote-eukaryote and prokaryote-prokaryote interaction amongst the strains may facilitate specific interactions with the host and may help to establish a niche within the host microbiota, underpinning the co-existence of different *Pseudovibrio* strains in the same host. A cohort of host-interactive systems has also been described in the recent genomic analysis of a new *Pseudovibrio* strain (Alex and Antunes, 2015), suggesting that the comparative data presented here will be representative of the *Pseudovibrio* genus, beyond the current collection of sequenced isolates.

## Conclusions

In this work we present 10 newly sequenced *Pseudovibrio* genomes, and we performed an extensive comparative genomic analysis including the two other publicly available genomes of strains FO-BEG1 and JE062. Our analyses indicate that bacteria belonging to the *Pseudovibrio* genus are versatile generalist bacteria well-adapted to thrive under different environmental conditions. We report the considerable diversity of systems potentially involved in the interaction with eukaryotes and with other bacteria, notwithstanding the fact that the majority of the *Pseudovibrio* strains were isolated from the same environment and are phylogenetically and metabolically related. Our data add a new perspective to the current knowledge on the *Pseudovibrio* genus, since we show that strain AD2 forms a clear divergent phylogenetic lineage. It is characterized by a considerable reduction in genome size and in systems described in other bacteria to be involved in the interaction with the host and its microbiota. This suggests that this strain might not colonize the host via attachment and penetration from the surrounding environment, but may in fact be vertically transmitted. Overall our analysis leads to the hypothesis that strain AD2 might represent the first example of a sponge-specific *Pseudovibrio*-lineage, being in an evolutionary stage of transition from a horizontally acquired to a vertically transmitted symbiont.

## Author contributions

SR, FG, and AD conceived the study. SC, OO, PC, FR, and CA prepared genomic DNA and submitted genomes for sequencing. SC, OO, PC, AF, FG processed the raw sequencing data and performed genome assembly. SR performed the comparative genomic analysis. CA performed the experimental work. SR, FR, AD and FG wrote the paper including comments of all co-authors.

### Conflict of interest statement

The authors declare that the research was conducted in the absence of any commercial or financial relationships that could be construed as a potential conflict of interest.

## Footnotes

^1^VelvetOptimiser Available at: http://bioinformatics.net.au/software.velvetoptimiser.shtml

^2^Sanger, Pfamscan Available at: http://www.ftp.com/ftp.sanger.ac.uk/pub/databases/Pfam/Tools/pfam_scan.pl

^3^ExPASy, Compute pI/Mw tool Available at: http://web.expasy.org/compute_pi/

## Supplementary material

Figure S1 **16S rRNA genes phylogenetic analysis**. Consensus phylogenetic tree constructed using the 16S rRNA genes of *Pseudovibrio* related bacteria. The values at the nodes indicate the bootstrap obtained during the reconstruction of the consensus tree performed with the build-in function in the ARB program. Strains investigated in the present work are indicated in bold red. Type stains are reported in bold black with the superscript “TS”.

Figure S2 **Distribution of the protein-coding genes of the 12 *Pseudovibrio* genomes amongst the COG**. J, Translation, ribosomal structure, and biogenesis; K, Transcription; L, Replication, recombination, and repair; B, Chromatin structure and dynamics; D, Cell cycle control, cell division, chromosome partitioning; T, Signal transduction mechanisms; M, Cell wall/membrane/envelope biogenesis; N, Cell motility; Z, Cytoskeleton; U, Intracellular trafficking, secretion, and vesicular transport; 0, Posttranslational modification, protein turnover, chaperones; X, Mobilome: prophages, transposons; C, Energy production and conversion; G, Carbohydrate transport and metabolism; E, Amino acid transport and metabolism; F, Nucleotide transport and metabolism; H, Coenzyme transport and metabolism; 1, Lipid transport and metabolism; P, Inorganic ion transport and metabolism; Q, Secondary metabolites biosynthesis, transport, and catabolism; R, General function prediction only; S, Function unknown; No COG, roteins not assigned to COG.

Figure S3 **Growth of *Pseudovibrio* isolates in minimal medium with KN03 as nitrogen source**. Data is the average of two biological replicates.

Figure S4 **Type 3 secretion system in the *Pseudovibrio* genus**. In **(A)** are reported the gene clusters coding for the T3SS identified in the *Pseudovibrio* genomes. Forward slashes separate genes identified in separate contigs. A representative T3SS gene cluster for *Pseudovibrio* was then used as query against all available GeneBank bacterial genomes, with the aim of searching homologous clusters. Only the best two hits of this analysis are shown in **(B)**. For all gene clusters, similar colors indicates homologous genes. In gray are reported homologous genes shared amongst the strains, but not identified as art of the T3SS structure with the approach we used.

Figure S5 **Rooted phylogenetic tree reconstructed using protein homologous to YscU belonging to the T3SS**. KEGG identifiers for each protein are reported. Only Bootstrap values higher than 50 are shown. Colors of the branches are based on the T3SS classification reported in Gazi et al. (2012). Flagellar proteins FlhB were used as out group.

Figure S6 **Type 4 secretion system in the *Pseudovibrio* genus**. In **(A)** are reported the gene clusters coding for the T4SS identified in the *Pseudovibrio* genomes. Forward slashes separate genes identified in separate contigs. A representative T4SS gene cluster for *Pseudovibrio* was then used as query against all available GeneBank bacterial genomes, with the aim of searching homologous clusters. Only the best two hits of this analysis are shown in **(B)**. For all gene clusters, similar colors indicates homologous genes. In gray are reported homologous genes shared amongst the strains, but not identified as part of the T455 structure with the approach we used.

Figure S7 **Type 6 secretion system in the *Pseudovibrio* genus**. In **(A,C)** are reported the gene clusters coding for the T6SS-I and the T6SS-II identified in the *Pseudovibrio* genomes. Forward slashes separate genes identified in separate contigs. Representative T6SS gene clusters for *Pseudovibrio* were then used as query against all available GeneBank bacterial genomes, with the aim of searching homologous clusters. Of this analysis, only the best two hits for T6SS-I and three hits for T6SS-II are shown in **(B,D)**, respectively. For all gene clusters, similar colors indicates homologous genes. In gray are reported homologous genes shared amongst the strains, but not identified as part of the T6SS structure with the approach we used.

Figure S8 **Unrooted phylogenetic tree reconstructed using protein homologous to lgIA belonging to the T6SS KEGG identifiers for each protein are reported**. Only Bootstrap values higher than 50 are shown. Color of the branches are based on the T6SS classification reported in Boyer et al. (2009).

Table S1 **Average nucleotide identity (ANI) and tetra nucleotide correlations calculated for the 12 *Pseudovibrio* strains. ANIb and ANIm refer to the ANI value calculated with the blast and mummer program, respectively**.

Table S2 **KEGG modules identified in the 12 *Pseudovibrio* genomes**.

Table S3 **Groups of potential T3SS effectors identified in the 12 *Pseudovibrio* genomes. Proteins were grouped according with their sequence similarity, domain architecture, and structural prediction**.

Table S4 **Type 5 secretion systems identified in the 12 *Pseudovibrio* genomes. Proteins were grouped according to their sequence similarity and domain architecture**.

Table S5 **Potential T6SS effectors and immunity proteins identified in the 12 *Pseudovibrio* genomes**.

Table S6 **List of potential toxin-like proteins encoded in the 12 *Pseudovibrio* genomes. Proteins were grouped according with their sequence similarity, and domain/repeat architecture**.

Table S7 **Proteins containing Rhs repeats, and SMI1/KNR4 and WHH domains encoded in the 12 *Pseudovibrio* genomes**.

Table S8 **List of Pfam domains used for the identification T3SS effectors in the *Pseudovibrio* genomes. These domains have been described to be present in T3SS effectors of other bacteria (Arnold et al., 2010)**.

Table S9 **List of domains used for the identification of toxin-like proteins in the *Pseudovibrio* genomes. This domains have been described to be present in bacterial toxins, and were obtained after a manual screening of the Pfam and TIGRFAM databases**.
