# Supplementary material for: Comparative Genomic Analysis Reveals a Diverse Repertoire of Genes Involved in Prokaryote-Eukaryote Interactions within the Pseudovibrio Genus
Source: Front Microbiol. 2016 Mar 30;7:387. doi: 10.3389/fmicb.2016.00387 (PMC4811931; doi:10.3389/fmicb.2016.00387)
Supplement: Figure S1 — 16S rRNA genes phylogenetic analysis. Consensus phylogenetic tree constructed using the 16S rRNA genes of Pseudovibrio related bacteria. The values at the nodes indicate the bootstrap obtained during the reconstruction of the consensus tree performed with the build-in function in the ARB program. Strains investigated in the present work are indicated in bold red. Type stains are reported in bold black with the superscript “TS”. [file Image1.PDF]

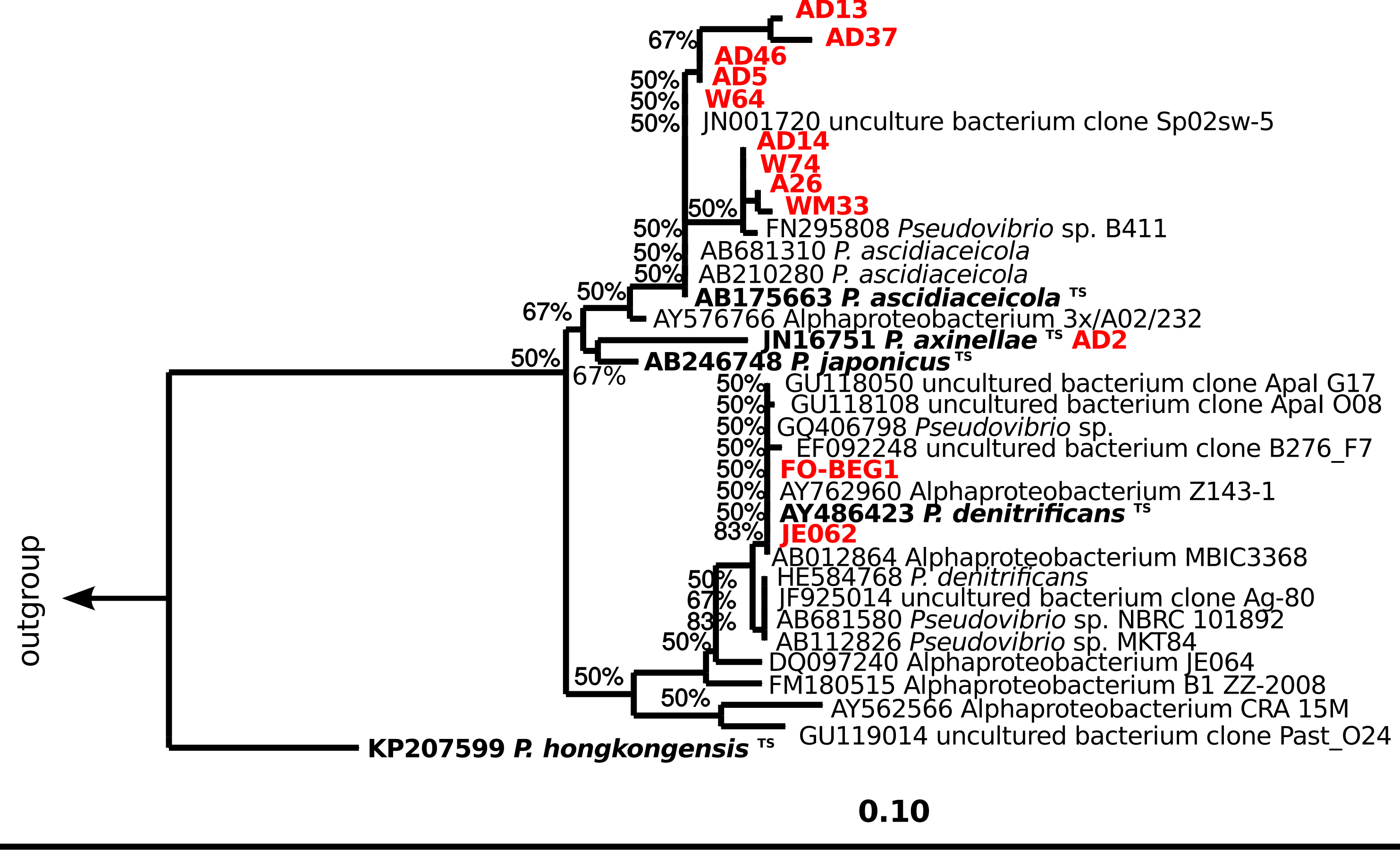


**Figure S1** 16S rRNA genes phylogenetic analysis. Consensus phylogenetic tree constructed using the 16S rRNA genes of *Pseudovibrio* related bacteria. The values at the nodes indicate the bootstrap obtained during the reconstruction of the consensus tree performed with the build-in function in the ARB program. Strains investigated in the present work are indicated in bold red. Type stains are reported in bold black with the superscript "TS"
