# Supplementary material for: Comparative Genomic Analysis Reveals a Diverse Repertoire of Genes Involved in Prokaryote-Eukaryote Interactions within the Pseudovibrio Genus
Source: Front Microbiol. 2016 Mar 30;7:387. doi: 10.3389/fmicb.2016.00387 (PMC4811931; doi:10.3389/fmicb.2016.00387)
Supplement: Figure S2 — Distribution of the protein-coding genes of the 12 Pseudovibrio genomes amongst the COG. J, Translation, ribosomal structure, and biogenesis; K, Transcription; L, Replication, recombination, and repair; B, Chromatin structure and dynamics; D, Cell cycle control, cell division, chromosome partitioning; T, Signal transduction mechanisms; M, Cell wall/membrane/envelope biogenesis; N, Cell motility; Z, Cytoskeleton; U, Intracellular trafficking, secretion, and vesicular transport; 0, Posttranslational modification, protein turnover, chaperones; X, Mobilome: prophages, transposons; C, Energy production and conversion; G, Carbohydrate transport and metabolism; E, Amino acid transport and metabolism; F, Nucleotide transport and metabolism; H, Coenzyme transport and metabolism; 1, Lipid transport and metabolism; P, Inorganic ion transport and metabolism; Q, Secondary metabolites biosynthesis, transport, and catabolism; R, General function prediction only; S, Function unknown; No COG, roteins not assigned to COG. [file Image2.PDF]

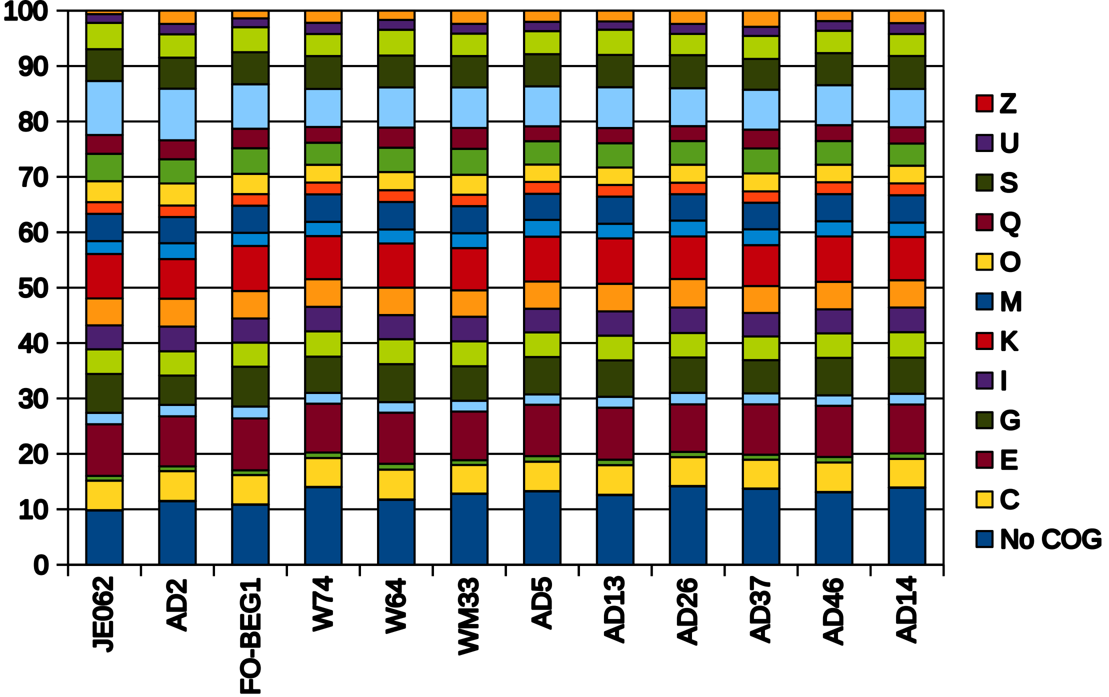

Figure S2 Distribution of the protein-coding genes of the 12 *Pseudovibrio* genomes amongst the COG.
J: Translation, ribosomal structure and biogenesis ; K: Transcription; L: Replication, recombination and repair; B: Chromatin structure and dynamics; D: Cell cycle control, cell division, chromosome partitioning; T: Signal transduction mechanisms; M: Cell wall/membrane/envelope biogenesis ; N: Cell motility; Z: Cytoskeleton; U: Intracellular trafficking, secretion, and vesicular transport ; O: Posttranslational modification, protein turnover, chaperones; X: Mobilome: prophages, transposons; C: Energy production and conversion; G: Carbohydrate transport and metabolism; E: Amino acid transport and metabolism; F: Nucleotide transport and metabolism; H: Coenzyme transport and metabolism ; I: Lipid transport and metabolism; P: Inorganic ion transport and metabolism ; Q: Secondary metabolites biosynthesis, transport and catabolism; R: General function prediction only ; S: Function unknown; No COG: proteins not assigned to COG.
