# Supplementary material for: Comparative Genomic Analysis Reveals a Diverse Repertoire of Genes Involved in Prokaryote-Eukaryote Interactions within the Pseudovibrio Genus
Source: Front Microbiol. 2016 Mar 30;7:387. doi: 10.3389/fmicb.2016.00387 (PMC4811931; doi:10.3389/fmicb.2016.00387)
Supplement: Figure S3 — Growth of Pseudovibrio isolates in minimal medium with KN03 as nitrogen source. Data is the average of two biological replicates. [file Image3.PDF]

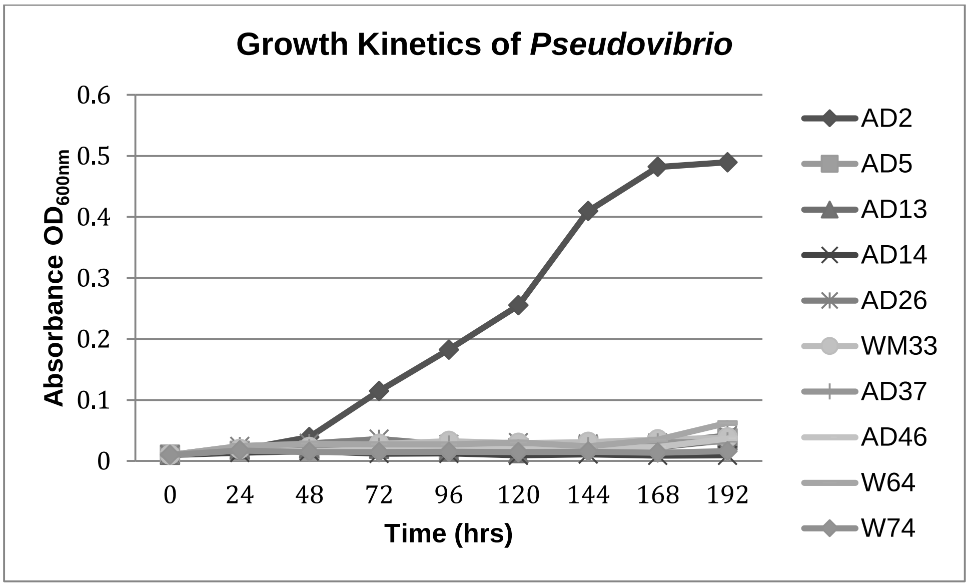

Figure S3. Growth of *Pseudovibrio* isolates in minimal medium with KNO3 as nitrogen source. Data is the average of two biological replicates.
