# Supplementary material for: Comparative Genomic Analysis Reveals a Diverse Repertoire of Genes Involved in Prokaryote-Eukaryote Interactions within the Pseudovibrio Genus
Source: Front Microbiol. 2016 Mar 30;7:387. doi: 10.3389/fmicb.2016.00387 (PMC4811931; doi:10.3389/fmicb.2016.00387)
Supplement: Figure S4 — Type 3 secretion system in the Pseudovibrio genus. In (A) are reported the gene clusters coding for the T3SS identified in the Pseudovibrio genomes. Forward slashes separate genes identified in separate contigs. A representative T3SS gene cluster for Pseudovibrio was then used as query against all available GeneBank bacterial genomes, with the aim of searching homologous clusters. Only the best two hits of this analysis are shown in (B). For all gene clusters, similar colors indicates homologous genes. In gray are reported homologous genes shared amongst the strains, but not identified as art of the T3SS structure with the approach we used. [file Image4.PDF]

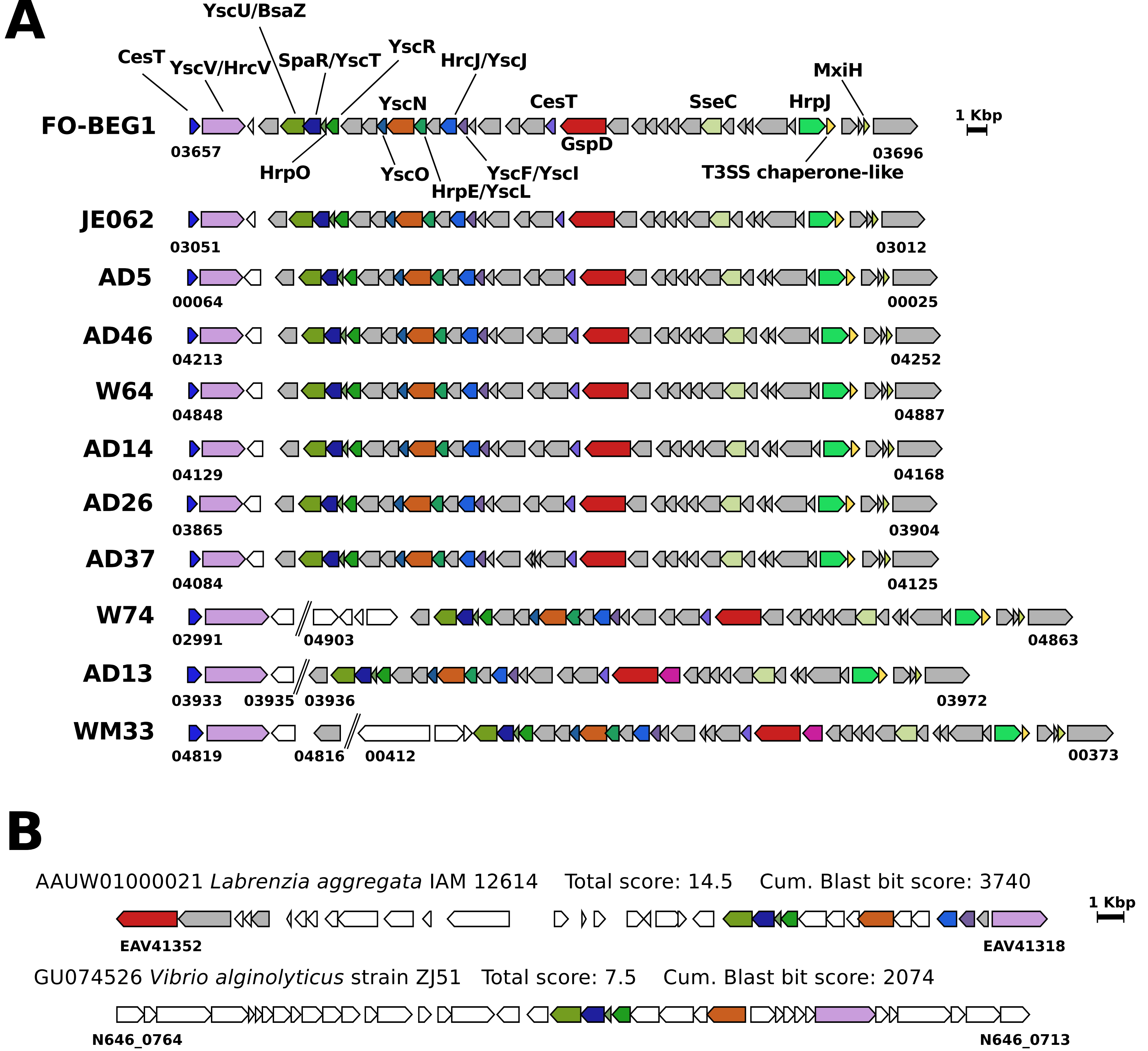

Figure S4 Type 3 secretion system in the *Pseudovibrio* genus. In **A** are reported the gene clusters coding for the T3SS identified in the *Pseudovibrio* genomes. Forward slashes separate genes identified in separate contigs. A representative T3SS gene cluster for *Pseudovibrio* was then used as query against all available GeneBank bacterial genomes, with the aim of searching homologous clusters. Only the best two hits of this analysis are shown in **B**. For all gene clusters, similar colours indicates homologous genes. In gray are reported homologous genes shared amongst the strains, but not identified as part of the T3SS structure with the approach we used.
