# Supplementary material for: Comparative Genomic Analysis Reveals a Diverse Repertoire of Genes Involved in Prokaryote-Eukaryote Interactions within the Pseudovibrio Genus
Source: Front Microbiol. 2016 Mar 30;7:387. doi: 10.3389/fmicb.2016.00387 (PMC4811931; doi:10.3389/fmicb.2016.00387)
Supplement: Figure S5 — Rooted phylogenetic tree reconstructed using protein homologous to YscU belonging to the T3SS. KEGG identifiers for each protein are reported. Only Bootstrap values higher than 50 are shown. Colors of the branches are based on the T3SS classification reported in Gazi et al. (2012). Flagellar proteins FlhB were used as out group. [file Image5.PDF]

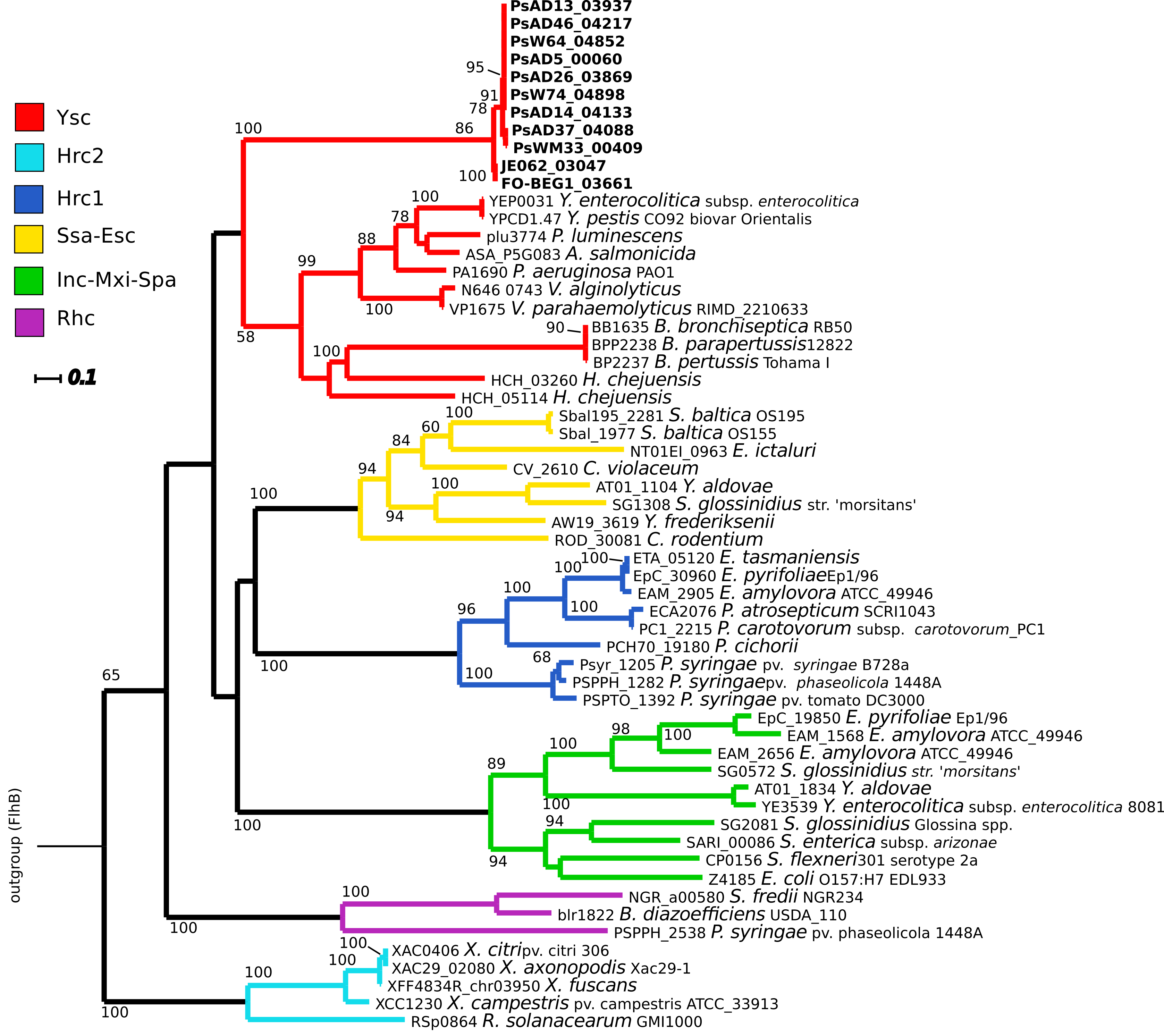

Figure S5 Rooted phylogenetic tree reconstructed using protein homologous to YscU belonging to the T3SS. KEGG identifiers for each protein are reported. Only Bootstrap values higher than 50 are shown. Colours of the branches are based on the T3SS classification reported in Gazi et al (Gazi et al., 2012). Flagellar proteins FlhB were used as outgroup.
