# Supplementary material for: Comparative Genomic Analysis Reveals a Diverse Repertoire of Genes Involved in Prokaryote-Eukaryote Interactions within the Pseudovibrio Genus
Source: Front Microbiol. 2016 Mar 30;7:387. doi: 10.3389/fmicb.2016.00387 (PMC4811931; doi:10.3389/fmicb.2016.00387)
Supplement: Figure S7 — Type 6 secretion system in the Pseudovibrio genus. In (A,C) are reported the gene clusters coding for the T6SS-I and the T6SS-II identified in the Pseudovibrio genomes. Forward slashes separate genes identified in separate contigs. Representative T6SS gene clusters for Pseudovibrio were then used as query against all available GeneBank bacterial genomes, with the aim of searching homologous clusters. Of this analysis, only the best two hits for T6SS-I and three hits for T6SS-II are shown in (B,D), respectively. For all gene clusters, similar colors indicates homologous genes. In gray are reported homologous genes shared amongst the strains, but not identified as part of the T6SS structure with the approach we used. [file Image7.PDF]

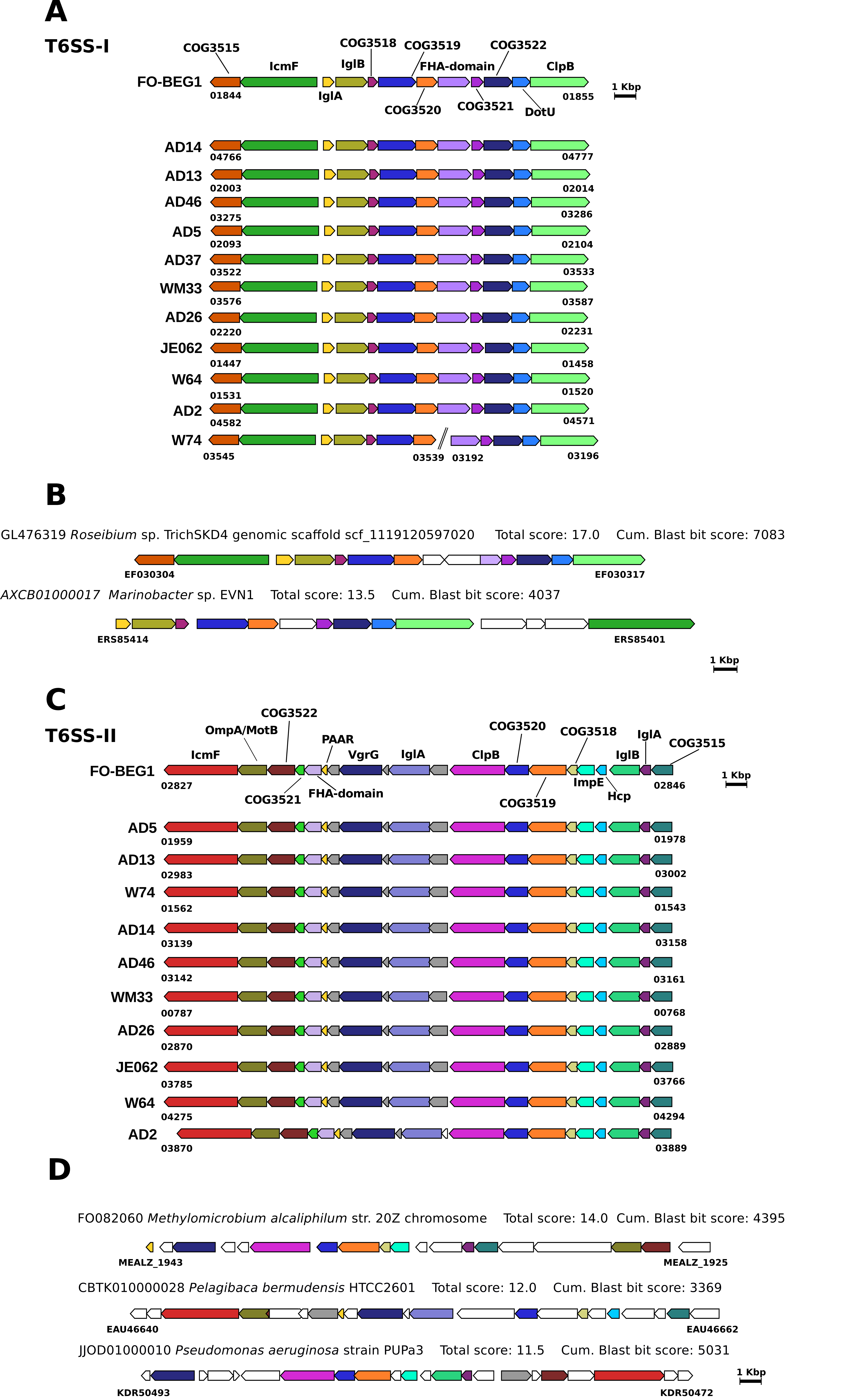

Figure S7 Type 6 secretion system in the *Pseudovibrio* genus. In **A** and **C** are reported the gene clusters coding for the T6SS-I and the T6SS-II identified in the *Pseudovibrio* genomes. Forward slashes separate genes identified in separate contigs. Representative T6SS gene clusters for *Pseudovibrio* were then used as query against all available GeneBank bacterial genomes, with the aim of searching homologous clusters. Of this analysis, only the best two hits for T6SS-I and three hits for T6SS-II are shown in **B** and **D**, respectively. For all gene clusters, similar colours indicates homologous genes. In gray are reported homologous genes shared amongst the strains, but not identified as part of the T6SS structure with the approach we used.
