# Supplementary material for: Comparative Genomic Analysis Reveals a Diverse Repertoire of Genes Involved in Prokaryote-Eukaryote Interactions within the Pseudovibrio Genus
Source: Front Microbiol. 2016 Mar 30;7:387. doi: 10.3389/fmicb.2016.00387 (PMC4811931; doi:10.3389/fmicb.2016.00387)
Supplement: Figure S8 — Unrooted phylogenetic tree reconstructed using protein homologous to lgIA belonging to the T6SS KEGG identifiers for each protein are reported. Only Bootstrap values higher than 50 are shown. Color of the branches are based on the T6SS classification reported in Boyer et al. (2009). [file Image8.PDF]

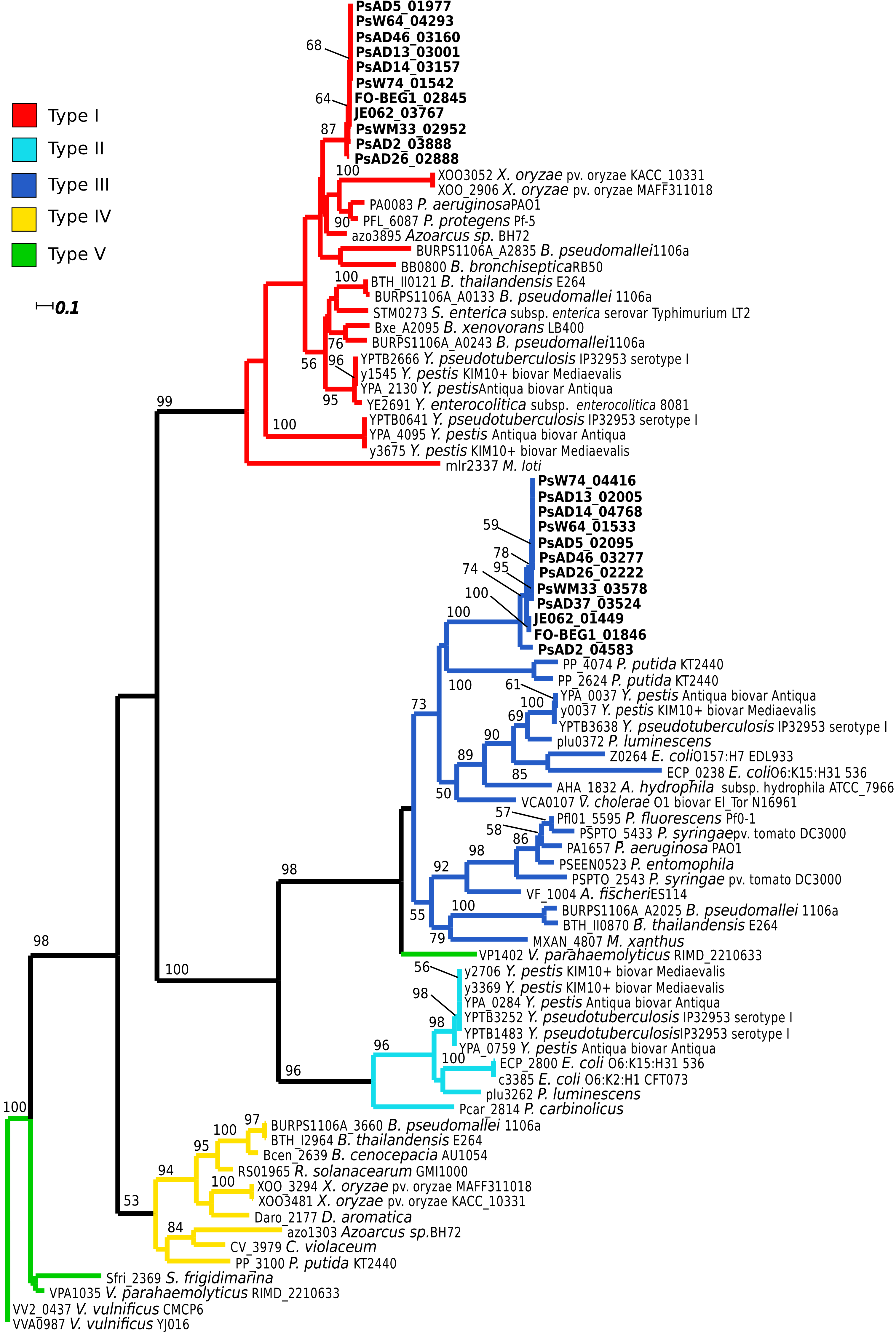

Figure S8 Unrooted phylogenetic tree reconstructed using protein homologous to IglA belonging to the T6SS KEGG identifiers for each protein are reported. Only Bootstrap values higher than 50 are shown. Color of the branches are based on the T6SS classification reported in Boyer et al. (Boyer et al., 2009).
